# Comparative Assessment of Whole Organ Tissue Processing Methods for the Isolation of Extracellular Vesicles From Intact Organs

**DOI:** 10.1002/jev2.70127

**Published:** 2025-09-03

**Authors:** Mohammed Tayab Hussain, Shani Austin‐Williams, Joel McCay, Hedayatullah Hayat, Thomas D. Wright, Marilena Christoforou, Ella Ykema, Mauro Perretti, Dianne Cooper, Andreas Margraf

**Affiliations:** ^1^ Centre for Biochemical Pharmacology William Harvey Research Institute Faculty of Medicine and Dentistry Queen Mary University of London London UK; ^2^ Centre for Haemato‐Oncology Barts Cancer Institute Faculty of Medicine and Dentistry Queen Mary University of London London UK; ^3^ Department of Anesthesiology, Intensive Care Medicine and Pain Therapy University Hospital Münster Münster Germany

**Keywords:** digest, disruption, extracellular vesicles, organ isolation

## Abstract

Extracellular vesicles (EVs) are small anuclear cellular membrane encapsulated fragments of importance for cellular interaction and transfer of information. These small vesicles, diverse in size and functionality, can be obtained from cells, tissues and bodily fluids. A complicated step for obtaining EVs from whole organs is understanding the optimal methodology for organ processing. In this study, we have examined two different techniques: one enzymatic and one novel non‐enzymatic automated tissue dissociation (ATD) machine. Animals were perfused, organs extracted, and techniques comparatively applied. We have used these techniques for organ‐based dissociation followed by EV isolation from the dissociated tissues (heart, kidney, lung). While both approaches allow isolation of intact EVs there are distinct differences in overall cell and particle yields. Our study highlights tissue specific inter‐organ variability and differential impact of dissociation strategies on organ‐based EV profiles, as well as cellular characteristics. Our findings indicate that EV yields and characteristics varies between enzymatic and ATD techniques as well as between organs with highest EV yield obtained from kidneys following enzymatic dissociation. Our findings can be rapidly transferred to other setups or developed to enable enumeration and characterization of EVs obtained from whole organs in physiological and pathological settings.

## Graphical abstract

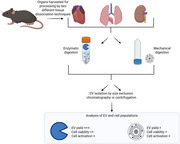

## 1 Introduction

Extracellular vesicles (EVs) are cell‐derived microstructures with diverse composition and a large variety of proposed functions and properties (Yanez‐Mo et al. 2015). The EV field is a rapidly expanding area of biology, with newly detected sub‐classes of EVs recently described (Anand et al. 2021; Zhang et al. 2021). Despite these new terminologies, EVs can be more broadly defined as lipid‐bilayer encapsulated particles that do not contain a nucleus and are therefore incapable of independent replication (Thery et al. 2018). The three main classes of EVs, exosomes, microvesicles/ectosomes, and apoptotic bodies, are separated due to distinct biogenesis pathways as well as their physico‐chemical features (Beaudoin and Grondin 1991; van der Pol et al. 2012). Exosomes are defined as the smallest in size, ranging from 20 to 100 nm in diameter (Muller et al. 2014; van der Pol et al. 2012). They are formed within the cell and are derived from intra‐luminal vesicles, through the endosomal recycling network and released as intact vesicles (van der Pol et al. 2012). Microvesicles, or ectosomes, are generated at the plasma‐membrane and are generally larger in size with diameters ranging from 100 to 1000 nm (van der Pol et al. 2012). Apoptotic bodies differ from the other two groups in that they are only generated during apoptosis and are considered the largest of the EV classes, with sizes ranging up to ∼5 µm in diameter (Elmore 2007).

EVs have been attributed a range of functions within the organism and are prominently reported to be involved in the shuttling of information between cells (Yanez‐Mo et al. 2015). They are released constitutively under basal conditions, but their phenotype and concentration changes when cells are exposed to stressors or mechanical challenge (Yates et al. 2022a, 2022b). All cells are thought to release EVs and numerous roles have been identified in both physiology and pathology (Deatherage and Cookson 2012; Hyenne et al. 2019; van der Pol et al. 2012); however, in vivo evidence for this is so far limited. Historically, EVs have been isolated from body fluids or cell culture (supernatant) preparations but this limits the ability to study the biology of EVs in the multicellular tissue‐context and from tissue resident cells. To overcome this limitation, focus is now shifting to tissue‐derived EVs.

Isolation of EVs from whole organs/tissues will provide a more physiologically representative EV population than those obtained from cells in culture, as well as further insight into intercellular EV‐based communication and shuttling occurring in health and disease (Crescitelli et al. 2020; Crescitelli et al. 2021). Notably, appearance and detection of EVs is context‐ and possibly also isolation technique‐dependent (Crescitelli et al. 2021). Thus, developing methodologies to study tissue‐derived EVs will aid our understanding of the function of these powerful nano‐organelles in vivo.

Methods for isolation of EVs involve ultrafiltration, centrifugation, and size exclusion chromatography, but obtaining and studying EVs from intact organs and tissues remains a challenge (Visan et al. 2022). Eukaryotic cells have a diverse range of properties and functions; thus, it is plausible that EV yields will differ between different organs. Equally, techniques for organ digestion are likely to influence efficiency and composition of isolated EVs. Importantly, cell viability can be impacted by mechanical or enzymatic digestion of tissues, thus affecting the overall EV yield and composition (Reichard and Asosingh 2019; Yaigoub et al. 2022). Application of distinct isolation methods must be thoroughly examined. The artificial release of EVs must be minimised and therefore conditions standardised to deliver adequate, reproducible, and reliable experimental outputs. To achieve this, protocols for EV isolation from fresh tissues need to be optimised to limit cell damage and ensure separation of EVs genuinely present in the original organ.

So far, only limited studies have been performed to assess isolation of EVs, and their yields, from various organs when different tissue processing techniques are applied (Huang et al. 2020; Matejovic et al. 2021). Recently, a rapid sterile tissue‐dissociation device, intended for generating single cell suspensions from tissue samples, was developed that avoids the use of enzymes, which require prolonged incubation times, potentially altering cell functionality, integrity or activation (Balamurugan et al. 2005; Scheuermann et al. 2019; Schmidt et al. 2018).

**FIGURE 1:**
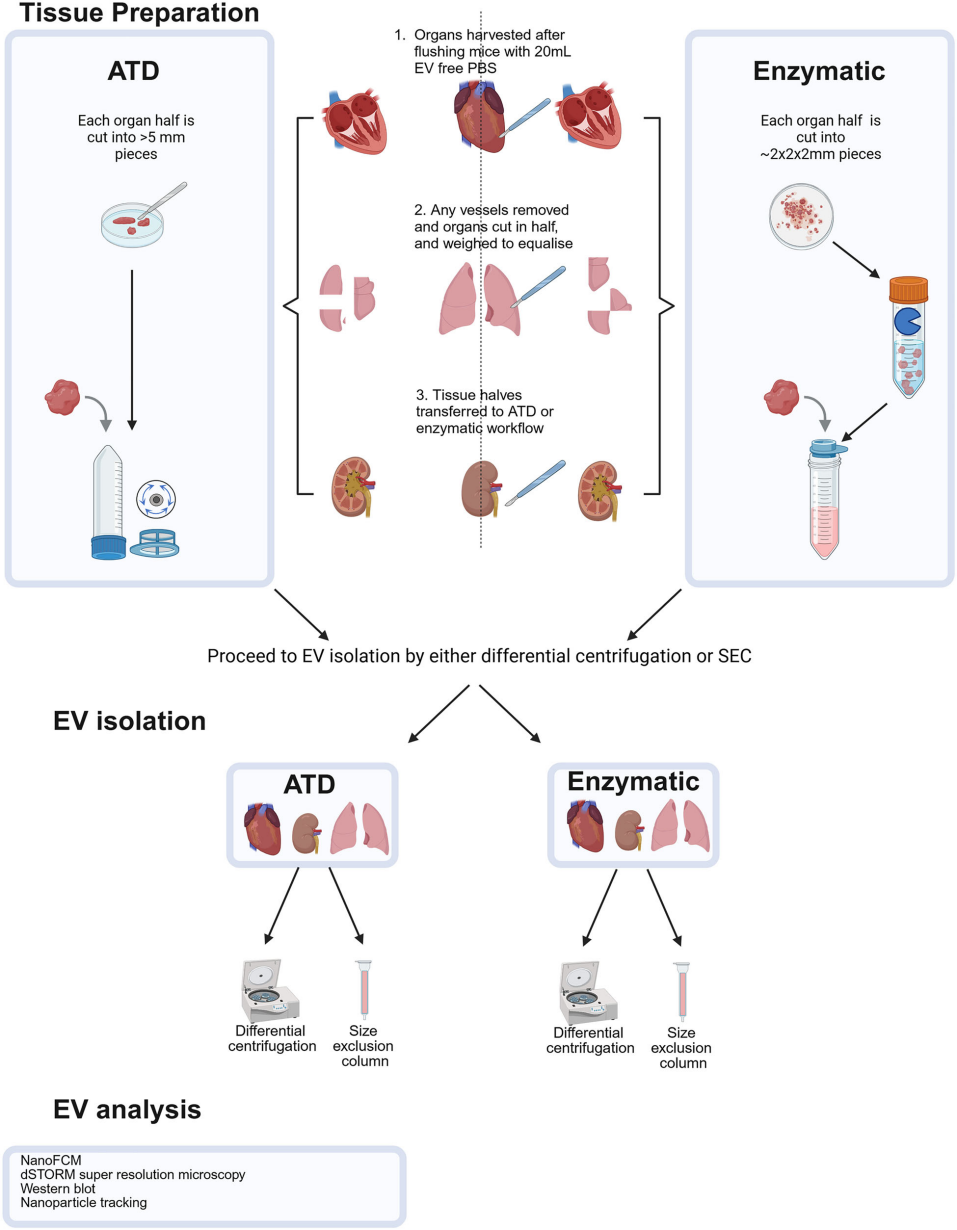
**Schematic overview of tissue preparation and EV isolation workflow**.

Here, we set out to compare the automated tissue dissociation (ATD) method with a more classical protocol of enzymatic organ digestion (Liang et al. 2023; Madissoon et al. 2023; Pircher et al. 2018), with the aim to determine potential differences in number and type of EVs obtained from various mouse tissues (Figure 1). Overall, both enzymatic and ATD approaches allow EV isolation from the tested organs with highest yields obtained from kidneys. When comparing preparation techniques, we conclude that enzymatic digestion results in higher EV yield, but characteristics vary across organs and isolation protocols. ATD can be utilised for tissue‐based EV purification, albeit with some adaptations. It remains evident that protocol optimisation and standardisation are required to enable reliable comparisons across different organ‐based EV studies.

## 2 Materials and Methods

### 2.1 Animals

KRN T cell receptor transgenic mice were maintained on a C57BL/6J background and housed under specific pathogen‐free conditions. Mice were kept under a 12‐h‐dark‐light cycle on standard chow pellet diet with ad libitum access to water. Eight 12–14‐week‐old male KRN C57BL/6J mice were used for comparative experimental analyses. All animal procedures were performed in accordance with institutional Animal Welfare Ethical Review Body and UK Home Office guidelines.

### 2.2 Organ Collection

Animals were sacrificed by exsanguination by cardiac puncture under isoflurane anesthesia. Organs were flushed with 20 mL room temperature EV‐free sterile double 0.22‐µm filtered Ca^2+^/Mg ^2+^ free PBS (PBS−/−) via the left ventricle of the heart (15 mL via the right ventricle and 5 mL via the left ventricle with clamping of the aorta to flush the coronary arteries). Flushing was performed at a flow rate of approximately 20 mL per minute to remove any remaining intravascular blood components. An anti‐erythrocyte antibody (Ter‐119) was later used to assess erythrocyte components within the EV fractions.

### 2.3 Organ Preparation

Heart, lungs, and kidneys were collected. Organs were weighed, cut into equal halves, weights of organ halves verified (Table S1), and each half processed by indicated methods (Figure 1). For enzymatic digestion, organs were cut with a scalpel blade on a cell culture dish into small (2 × 2 × 2 mm) pieces. The tissue pieces were then transferred into 2.5 mL of pre‐warmed digestion media, consisting of HBSS + Ca/Mg, 0.2 mg/mL Liberase TH (Merck–Roche #5401151001) and 60 U/mL DNase 1 (Merck‐Roche #10104159001) (Mateescu et al. 2017). Samples were incubated for 30 min at 37°C with agitation (200 rpm). After 15 min, organs were triturated using an 18 g needle, followed by continuation of the incubation interval. After a 30‐min incubation time, organs were again triturated and passed through a 70 µm cell strainer. Two hundred microlitres of EV‐free foetal bovine serum was added to quench enzymes (Gibco # A2720801). The cells were then pelleted by centrifugation at 1200 × *g* for 15 min at 4°C and supernatant used for further EV analysis.

For the ATD protocol, a TissueGrinder machine (Fast Forward Discoveries) was utilized. Organs were cut with a scalpel into large (>5 mm) pieces and placed into commercially available, custom‐made grinding‐inserts, containing a 70 µm cell strainer. PBS−/− was added (1 mL). ATD protocols were chosen according to manufacturer recommendations (Table 1). Following the grinding process, samples were filled up to 2.5 mL, 200 µL EV‐free foetal bovine serum was added and samples were centrifuged at 1200 × *g*, 15 min, 4°C to pellet cells.

**TABLE 1:** Automated tissue disintegrator settings.

Organ/Tissue	Total duration	Duration of steps	Number of steps	Speed	Filter size
Heart	3 min 10 s (with acceleration times: 3 min 53 s)	10–30 s	10	10–70 rpm	70 µm
Kidney	3 min 00 s (with acceleration time: 3 min 31 s)	20–30 s	7	12–25 rpm	70 µm
Lung	1 min 40 s (with acceleration time: 2 min 02 s)	10–30 s	6	10–18 rpm	70 µm

### 2.4 EV Preparation and Labelling

For collection of EV fractions, both enzymatic and ATD supernatants were centrifuged in 1.5 mL tubes at 4400 × *g*, 4°C for 15 min to pellet remaining cells. The supernatant was collected, transferred to new 1.5 mL tubes and centrifuged at 13,000 × *g*, for 2 min at 4°C, to pellet apoptotic bodies, which was repeated once (Oggero et al. 2021). EV isolation was performed using two separate methodologies.

#### 2.4.1 Differential Centrifugation

Differential centrifugation for isolation of large and intermediate EVs. EV supernatants were centrifuged at 20,000 × *g* at 4°C for 30 min to pellet EVs. After aspiration, pelleted EVs were resuspended in 1 mL PBS−/− for aliquoting prior to freezing (100 µL aliquots) and stored at −80°C until use. Centrifugation at 20,000  × *g* is sufficient to pellet larger EVs such as microvesicles. However, smaller EVs will also be pelleted at 20,000 × *g*, thus this fraction is determined as *enriched* in larger vesicles, although the population is truly heterogenous (Witwer et al. 2013). This sample, enriched in microvesicles will be described as EVs throughout this study in concordance with recent developments in the field (Thery et al. 2018). Prior to analysis, frozen EVs were thawed rapidly at 37°C and 7 µL sample was diluted with 23 µL EV‐free double filtered PBS−/−.

#### 2.4.2 Size Exclusion Chromatography (SEC) for Isolation of Small EVs

##### 2.4.2.1 SEC Preparation

Chromatography columns (BioRad Econo‐Pac Chromatography columns, Cat #7321010) were loaded with 14 mL sepharose CL‐2B (GE Healthcare, Sepharose Agarose Gel Filtration Media, Cat #10217754) followed by 10 mL EV‐free double filtered PBS−/− and left overnight at 4°C. The next day the upper bed support was placed on top of the sepharose and the column was washed twice with 10 mL PBS−/−. A final wash was performed with 10 mL PBS−/− with 0.03% Tween 20.

##### 2.4.2.2 EV Isolation

Following the final wash step and elution of all the wash buffer, 1 mL of sample was loaded onto the column. Sample in this context refers to the supernatants of organ homogenates after one centrifugation at 4400 × *g* followed by two centrifugations at 13,000 × *g*, all steps were carried out at 4°C as described above. 10 mL PBS−/− with 0.03% Tween 20 was then added to the column and 20 fractions of approximately 500 µL were collected for each sample.

In all cases, each column was reused once for a second sample of the same organ and preparation protocol (i.e., enzymatic or ATD). Columns were washed as described above prior to reuse.

##### 2.4.2.3 Sample Concentration

Amicon Ultra Centrifugal Filters, 10 kDa MWCO (Millipore, Merck) were prepared by washing columns with PBS−/− followed by two rounds of milli‐Q ultrapure water. Columns were centrifuged at 4000 × *g* for 10 min after each addition. EV enriched fractions from SEC isolation were pooled and loaded onto the columns and centrifuged for 10 min at 4000 × *g*. Once the sample had passed through, the columns were washed with 14 mL PBS−/− and centrifuged at 4000 × *g* for 30 min. Concentrated EVs were then retrieved from the column filter.

### 2.5 NanoFCM

Fluorescently labelled EVs were quantified by high resolution flow cytometry using a Flow NanoAnalyzer Instrument (NanoFCM, Inc.). For NanoFCM analysis, samples isolated via differential centrifugation were incubated with the indicated antibody diluted 1:30 (final concentration 6.7mg/mL) or 1.67 nM CFSE (5(6)‐CFDA, SE; CFSE (5(and‐6)‐Carboxyfluorescein Diacetate, Succinimidyl Ester; Invitrogen; cat: C1157) for 30 min at 4°C and protected from light. Antibodies used for EV labelling were as follows: anti‐CD9 (clone MZ3; Biolegend; cat:124812); anti‐Ter119 (clone TER‐119; Biolegend; cat:116212); anti‐CD31 (clone 390; eBiosciences; cat:12‐0311‐82) and anti‐CD326 (clone G8.8; Biolegend; cat:118214). EV solutions were then washed to remove unbound dye with PBS−/− and centrifuged again at 20,000 × *g* for 30 min for large and intermediate EVs. Following centrifugation, supernatants were aspirated, and EV pellets resuspended in PBS−/− with a target concentration of 2000–12,000 events in total for analysis by NanoFCM (Figures S1–S3). Following acquisition, samples were incubated with 0.1% Triton (final) for 20 min and the remaining particles subtracted from EV counts to exclude non‐vesicular particles (Brennan et al. 2020; Liu, Tian, et al. 2022; Tian et al. 2020).

For SEC samples, EVs were incubated with the indicated antibody diluted 1:30 (final concentration 6.7 mg/mL) or 1.67 µM BODIPY FL Maleimide (BODIPY FL N‐(2‐Aminoethyl)) Maleimide; Invitrogen; cat: B10250) for 30 min at 4°C and protected from light. Antibodies used for EV labelling were as follows: anti‐CD9 (clone MZ3; Biolegend; cat:124812); anti‐Ter119 (clone TER‐119; Biolegend; cat:116212); anti‐CD31 (clone 390; eBiosciences; cat:12‐0311‐82); anti‐CD326 (clone G8.8; Biolegend; cat:118214); anti‐CD41 (clone eBioMWReg30 (MWReg30); Invitrogen; cat: 17‐0411‐82) and anti‐cardiac troponin T (clone REA400; Miltenyi Biotec; cat: 130‐119‐575). Fluorescently labelled samples were then diluted with PBS−/− to achieve a final antibody concentration of 5.6 ng/mL, or 1.39 nM BODIPY, before being acquired on the NanoFCM.

Samples isolated via both SEC and differential centrifugation were acquired on the NanoFCM with the same settings and calibration steps. All experiments were carried out with suitable controls including: fluorescence minus one (FMO) controls for EVs stained with two markers, antibody and dye only controls and unstained EVs (Figure S4) (Brennan et al. 2020; Liu, Tian, et al. 2022; Tian et al. 2020).

The NanoFCM NanoAnalyzer was calibrated using dual‐laser quality control beads (NanoFCM, Inc.) and Silica Nanospheres Cocktail #1 (65–155 nm; NanoFCM, Inc.) in order to standardise particle concentration and size, respectively. An aliquot of the preferential solvent for EV samples was acquired as blank control. Quality control beads, sizing beads and fluorescently labelled EVs were all acquired on the NanoAnalyzer with a sampling pressure of 1.0 kPa and events recorded for a total of 1 min.

### 2.6 NanoSight NTA

For comparative assessment a nanoparticle tracking approach was chosen. Prepared solutions containing EVs were diluted to an appropriate concentration based on pre‐tests for running on the NanoSight. EV samples were loaded onto the NanoSight NS300 (Malvern Instruments Ltd., Malvern, UK) using a 1 mL syringe. At least 200 µL of each sample were flushed through the NanoSight before attaching the syringe pump. Three 60s videos were obtained for each sample, including sterile, double filtered PBS−/− ‘blank’ control using NTA 3.4 software—camera level: 11/12. Settings for analysis were as follows: Detection threshold: 3–4, blur size: auto. Matched samples were run using the same focus and camera level and analysed using the same detection threshold where possible, ensuring the brightness was consistent between samples. The following dilutions were applied: Kidney 1:200; Lung 1:100; Heart 1:50.

### 2.7 Flow Cytometry

Flow cytometry analysis of intact cells was performed on a BD Fortessa instrument equipped with four lasers. Cells were obtained as aliquots from the same samples used for EV determination, incubated for 30 min at 4°C with the following antibodies: anti‐CD41 (clone eBioMWReg30; from eBioscience; cat: 17‐0411‐80); anti‐GPIIb/IIIa (clone: Jon/A; Emfret analytics); anti‐CD45 (clone REA737; Macs Miltenyi Biotec; cat: 130‐110‐665); anti‐CD62L (clone MEL‐14; biolegend; cat: 104438); anti‐CD62P (clone RB40.34; BD Biosciences; cat: 564289) and a live‐dead marker (eBioscience Fixable Viability Dye eFluor780; ThermoFisher Scientific). Analysis was performed using FlowJo V10 software. Gating was applied (Figure S5) to either obtain overall viable cells out of total events or focused on CD45+ events. Viability measurements were performed according to reagent instructions. Percentage of dead cells within CD45+ was obtained by prior gating on CD45+ events. Gating on CD45 followed by gating on CD41 was used to obtain fractions of %CD41+ out of CD45+ cells. Expression of CD62L was obtained as Median Fluorescence Intensity (MFI) out of CD45+ cells, whereas P‐selectin (CD62P) and GPIIb/IIIa MFIs were obtained from CD41+ single cells.

### 2.8 Oni Super Resolution Microscopy

Super resolution images of EVs were acquired using an Oxford Nano Imager (ONI) dSTORM microscope (Nanoimager S, Oxford Nanoimaging, UK). Samples were thawed to room temperature and kept in a humidity chamber once fixed and stained prior to analysis. Machine calibration was performed to ensure accurate channel mapping and sizing using pre‐prepared bead slides commercially produced and validated by ONI using 100 nm Tetraspek microspheres (Invitrogen T7279). Beads and samples were imaged using a 100x oil objective lens and the microscope was warmed to between 26 and 32°C prior to use to minimise sample drift. A pre‐determined light program was installed for the Pan membrane and Pan tetraspanin antibodies as per ONI guidelines. Laser powers, frames and target antibodies can be seen in Table 2. Camera exposure was set to 40 ms and frequency 25 Hz. 3–5 fields of view were taken per lane (each sample) to give an average total cluster number staining positive for the pan membrane stain, which equates to a single EV. Super resolution and single molecule localization methods can be seen in Table 3.

**TABLE 2:** dSTORM Oni settings.

Laser	Power (mW)	Channel number	Frames acquired	Antibody
488	180	2	3000	Pan membrane
561	36.5	0	1000	Pan tetraspanin
647	170	1	1000	N/A

**TABLE 3:** dSTORM imaging paramented for filtering localisations, cluster filtering and counting tools.

Parameter	Range
Frame index	0–3999 (Channel 0 = 0–999, channel 1 = 1000–1999, Channel 2 = 2000–3999).
Photon count	200–7500
Sigma	50–225 nm
Localisation precision	0–10 nm
TIRF angle	52°
Z lock focus range	−400 to −200 um
Numerical aperture	1.4NA
Circularity	0.5–1
Area	700–1,000,000 nm^2^
Cluster distance, eps	85 nm
Exposure	40 ms

The light programme was run as channel 0, 1 and 2 with the frame index per channel below. Channels 0 and 2 were isolated only for characterising pan membrane markers (488 nm) and Pan tetraspanin markers (561 nm), respectively. The ONI generated EV profiling programme “AutoEv detection” for these specific stains was used. DBSCAN filtering and counting tool specifics for each algorithm can be found at: https://oni.wpenginepowered.com/wp‐content/uploads/2024/05/EV‐Profiler‐NimOS‐Updated‐May‐6_2024.pdf.

Data were analysed on the CODI platform using the EV profiling algorithm for Pan membrane and Pan tetraspanin antibodies. DBSCAN algorithm analysis was applied to improve clustering sensitivity and remove background noise. Clusters were counted and analysed for localisations of antibody > 5 and only channels 0 and 2 (lasers 488 and 561 nm) were counted as channels 1 and 3 did not correspond to positive pan membrane or tetraspanin blinking. Batch analysis was applied to all samples before CSV files were uploaded to prism graph pad for analysis and graph production.

### 2.9 Cryo Electron Microscopy

Electron Microscopy (EM) 300 copper mesh grids were treated with glow‐discharge using a Harrick plasma cleaner for 45 s at the MED RF power level. Subsequently, a 4 µL EV suspension was added to the top of EM grids. EM grids were blotted for 2.5 s under 95% humidity and rapidly frozen using a Leica GP2 plunger. A Jeol JEM‐2100+ electron microscope with a Gatan OneView 16MP camera was used to collect images from the EM grids. The microscope operated at an accelerating voltage of 200 kV; SerialEM software was employed to acquire digital images in low‐dose mode. Images were captured at a magnification of 50k. Defocus range was set at −2.5 to −5 µm.

### 2.10 Western Blot Analysis

EVs were isolated from tissues as described above and lysed in RIPA buffer (Thermofisher Scientific) with 1% protease inhibitor (Thermofisher Scientific). Total protein was quantified using a microBCA assay (Boster Bio) according to the manufacturer's instructions. Sample volumes were adjusted to ensure equal protein loading of gels and loaded onto a 10% polyacrylamide NuPAGE mini protein gel (Thermofisher Scientific) alongside a Precision Plus Protein Standard ladder (Bio Rad cat # 161–0373). Following electrophoresis proteins were transferred to activated PVDF membranes by wet transfer in tris/glycine buffer (BioRad #1610771) containing 20% ethanol. Membranes were then stained for total protein with TotalStain Q (Azure Biosytems). Membranes were then blocked (5% non‐fat milk in PBS containing 0.05% Tween (PBS‐T), washed and incubated with primary antibodies in 5% w/v non‐fat milk in PBS‐T overnight. Appropriate secondary horseradish peroxidase (HRP)‐conjugated antibodies (goat anti‐rabbit HRP, P0448, Dako; final concentration 1 µg/mL and goat anti‐mouse HRP, P0447, Dako; final concentration 1 µg/mL) were used for incubation and a Pierce ECL Western Blotting Substrate (Thermofisher Scientific) was added for detection. Images were captured in a Gel Doc system (FluorChem E). The following antibodies were used: Calnexin (AF18, ThermoFisher; final concentration 1 µg/mL); CD63 (2H5I1, ThermoFisher; final concentration: 0.44 µg/mL); Flotillin‐1 (133497, Abcam; final concentration 0.05 µg/mL); Apolipoprotein A1 (300085, Abcam; final concentration 0.5 µg/mL).

### 2.11 Statistical Analyses

All statistical analyses and graphing were performed using GraphPad Prism 8 Software and FlowJo V10 for flow cytometry plots. Data are expressed as mean ± standard error of the mean (SEM). For comparison of two groups a students unpaired *t*‐test was applied. For multiple groups an ANOVA with posthoc analysis was applied. Details on used statistical methods and group sizes are indicated in each figure legend. A *p* ≤ 0.05 was considered statistically significant.

## 3 Results

### 3.1 EV Profiles Differ Between Organs

**FIGURE 2:**
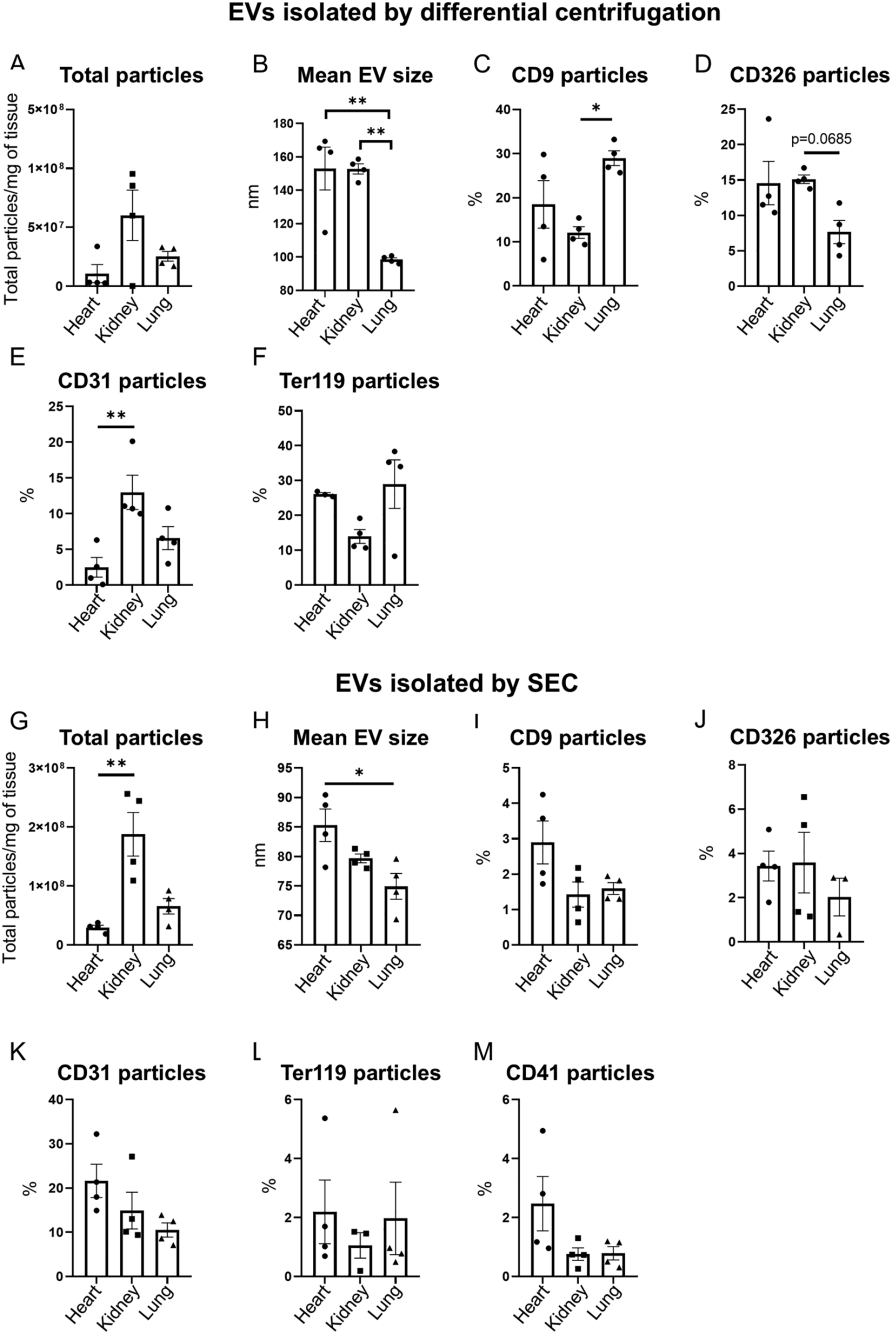
**Organ‐based detection of extracellular vesicles**. An enzymatic digestion approach was chosen to compare EV separation and characterization between three different organs (murine heart, kidney and lung). Measurements were performed using a NanoFCM particle subset enumeration. Data are shown for EVs isolated by differential centrifugation (A‐F). (A) Total CFSE+ particle concentration per mg of tissue. (B) EV size across the organs analysed. (C) Percentage of CD9+ particles out of all CFSE+ particles. (D) Percentage of CD326+ particles out of all CFSE+ particles. (E) Percentage of CD31+ particles out of all CFSE+ particles. (F) Percentage of Ter119 particles out of all CFSE+ particles. Data are shown for EVs isolated by SEC (G‐M) (G) Total BODIPY+ particles per mg of tissue. (H) EV size across the organs analysed. (I) Percentage of CD9+ particles out of all BODIPY+ particles. (J) Percentage of CD326 particles out of all BODIPY+ particles. (K) Percentage of CD31 particles out of all BODIPY+ particles. (L) Percentage of Ter‐119 particles out of all BODIPY+ particles. (M) Additional staining of CD41+ particles out of all BODIPY+ particles within the SEC processed samples. Data are mean ± SEM, *n* ≥ 3 per group. One‐way ANOVA with Tukey's multiple comparisons test or Kruskal–Wallis test with Dunn's multiple comparisons test; Brown‐Forsythe test was performed for equality of variances; *p < 0.05, **p < 0.01.

EVs can be obtained readily from cells grown in culture, but also from parts of intact tissues, as recently demonstrated (Bojmar et al. 2021; Crescitelli et al. 2021; Hurwitz et al. 2019). Studies reporting EV isolation from tissue sections of organs have used various methodologies to disrupt the tissue and recover EVs (Claridge et al. 2021; Crescitelli et al. 2021; Liu, Jin, et al. 2022; Zieren et al. 2020). Distinct differences in organ composition need to be considered when assessing tissue‐specific EV yield. To address inter‐organ variability of EV recovery, we first focused on the comparison of EV profiles from heart, kidney, and lung following standardised enzymatic digestion (liberase‐based). EV counts and mean diameter were quantified using a NanoFCM. Absolute EV counts as well as size distribution varied between each organ. Large and intermediate EVs recovered by differential centrifugation were highest in kidneys (Figure 2A). EVs recovered from the heart and kidney were of comparable size, while those recovered from the lung were significantly smaller (Figure 2B). Total particle count was also quantified by Nanoparticle tracking analysis with the greatest yield of particles observed in heart digests (Figure S6A).

The percentage of EVs isolated by differential centrifugation, positive for the tetraspanin CD9 was highest in the lung and significantly lower in kidney samples (Figure 2C). In contrast, numbers of particles positive for the cell specific markers CD326 (EpCAM; epithelial cells) were slightly higher (Figure 2D), and for CD31 (endothelial cells/platelets) significantly higher (Figure 2E), in kidney EVs compared to lung or heart EVs, respectively. Numbers of Ter‐119 positive particles (erythrocytes) were not significantly different between the organs tested (Figure 2F).

Since isolation methods of EVs differentially impact size and phenotypic profiles, we repeated these analyses using size exclusion chromatography (SEC) to focus on smaller EV fractions. Again, EV counts as well as size distribution varied between organs with highest number of small EVs again detectable in the kidney (Figure 2G). Mean EV size was expectedly reduced in SEC samples (between 60 and 100 nm) compared to differential centrifugation‐based large‐intermediate EV isolation, and again EVs recovered from the lung were smaller than those recovered from the heart and kidney (Figure 2H). Assessment of CD9 (Figure 2I), CD326 (Figure 2J), CD31 (Figure 2K), Ter119 (Figure 2L) and CD41 (Figure 2M) showed no significant differences between the organs. EV purity was assessed as percentage of positively (CFSE/BODIPY) labelled particles out of total detected particles by NanoFCM. Purity was highest in kidney samples following isolation of EVs by differential centrifugation (Figure S6B), whereas no differences between organs were observed when EVs were isolated by SEC (Figure S6C).

### 3.2 EVs Can be Extracted From Whole Organs by Both Enzymatic and Tissue Disruption Methods With Varying Yields

**FIGURE 3:**
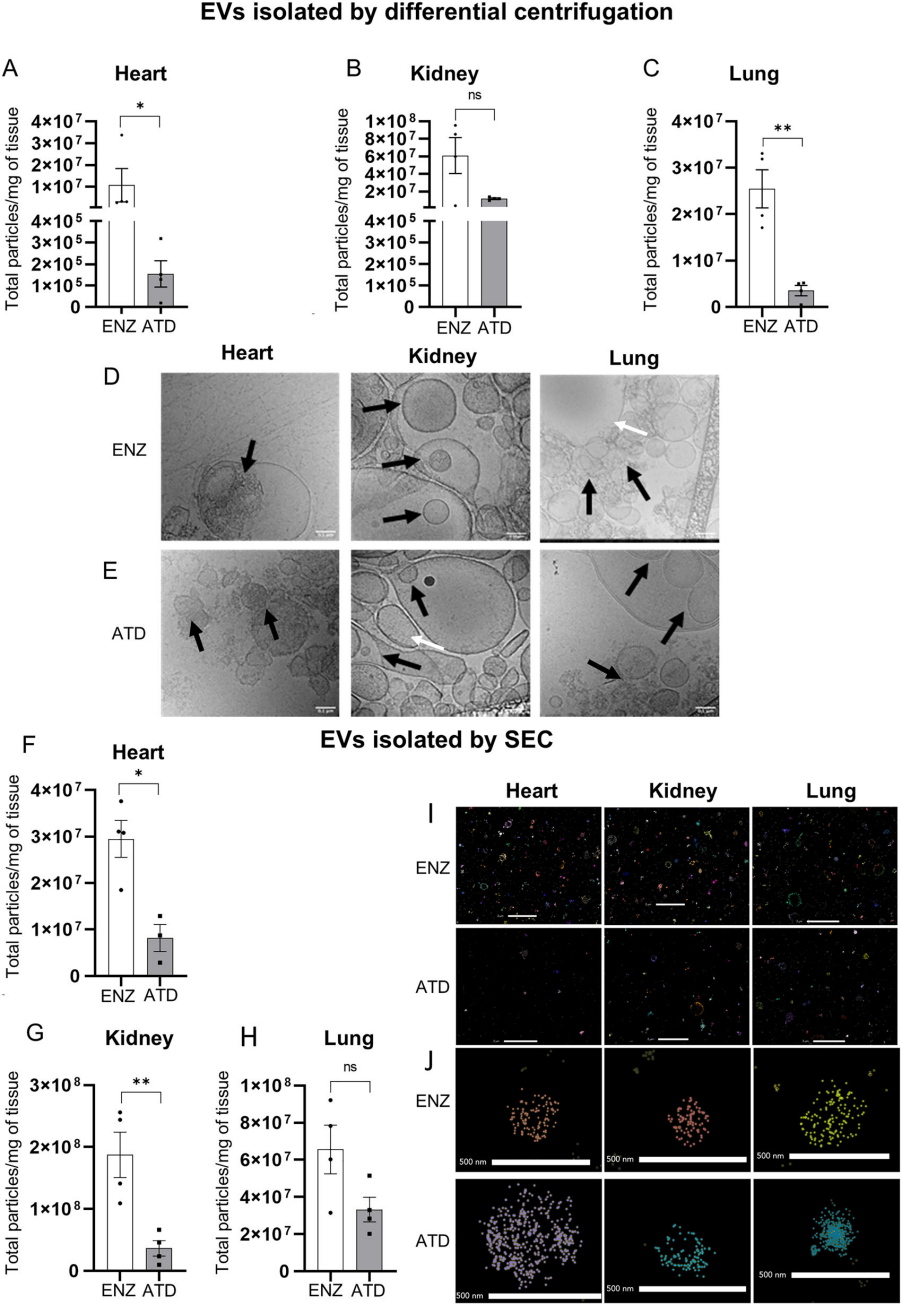
**EVs are extracted from both enzymatic and mechanical tissue dissociated organs**. Data are shown for EVs isolated by differential centrifugation (A–E) and SEC (F–H). NanoFCM based enumeration of CFSE+ total particles, isolated by differential centrifugation, per mg tissue in enzymatically or automated tissue dissociator (ATD) processed samples: (A) heart (B) kidney and (C) lungs. (D, E) Exemplary cryo‐electron microscopy images of organs. Black arrows indicate areas depicting electron‐dense or multi‐layered EVs and white arrows highlight clear EVs. NanoFCM based enumeration of total particles, isolated by SEC, per mg tissue in enzymatically or ATD processed samples: (F) heart, (G) kidney and (H) lungs. (I, J) EVs isolated by SEC analysed by dSTORM super resolution microscopy. EVs were captured onto an EV profiler 2 chip using phosphatidylserine and stained with ONI pan membrane antibody. Data are mean ± SEM, *n* ≥ 3 per group. Unpaired *t*‐test; * indicates *p* < 0.05, ** indicates *p* < 0.01.

Using a centrifugation‐based isolation method for large and intermediate EVs, we next compared an established enzymatic protocol with a novel, adaptable, commercially available and automated non‐enzymatic tissue‐dissociation protocol developed for the generation of single cells (Table 1), to prepare EVs from murine kidneys, hearts and lungs (Scheuermann et al. 2022; Soteriou et al. 2023). Total large to intermediate EVs were significantly higher in enzymatic digests of hearts (Figure 3A), slightly elevated in enzymatically digested kidneys (Figure 3B) and significantly elevated in enzymatic digests of lungs (Figure 3C). Absolute particle counts per mg of tissue were confirmed using the NanoSight with significantly higher numbers observed in enzymatic digests of heart and lung than samples prepared using the ATD (Figure S7A).

Cryo‐electron microscopy of samples showed heterogenous vesicles and particles of different sizes (Figure 3D,E). Both clear and electron‐dense vesicles (Yuana et al. 2013) could be observed in the enzymatic as well as the ATD processed samples. Similarly, multilayered vesicles could be noted. EM imaging demonstrated presence of large vesicles which due to technical limitations may not be adequately detected by either the NanoFCM or NanoSight NTA.

We next performed SEC‐based isolation of EVs and quantified total small EVs per mg of tissue. Small EV yield was again higher in the enzymatic group in hearts (Figure 3F), kidney (Figure 3G) and lung (Figure 3H). EV purity was again assessed as percentage of positively (CFSE/BODIPY) labelled particles out of total detected particles by NanoFCM (Figure S7B,C). Next, we used dSTORM super‐resolution microscopy as a further analytical approach. EV measurements using a proprietary Oni pan‐membrane antibody verified presence of different EV clusters (Figure 3I). Single EV representations following DBSCAN clustering confirmed presence of distinct EV preparations according to organ and dissociation strategy (Figure 3J). ATD samples tended to have a smaller number of positive clusters for EV membrane staining compared to enzyme samples (Figure S7D). Kidney samples prepared by enzymatic digestion had the highest number of positive clusters for EV membrane staining, followed by lung and heart samples, which correlated with yields determined by NanoFCM (Figure S7E, technical replicates). Cluster size was smaller in samples isolated by ATD compared to enzyme across all samples (Figure S7F).

### 3.3 EV Profile Differs According to the Use of Different Dissociation Techniques

**FIGURE 4:**
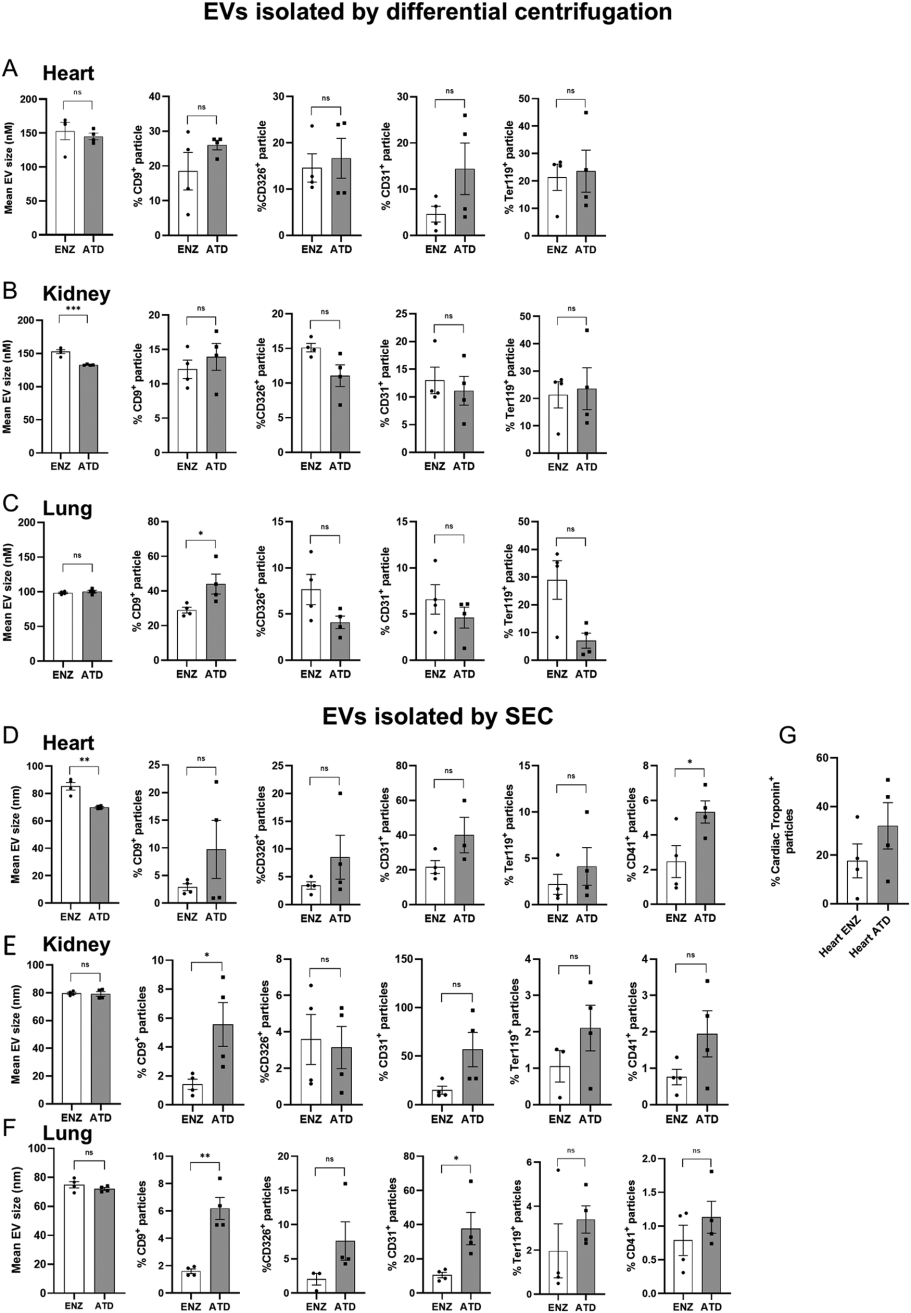
**Organ‐specific fractional attribution of EV populations**. (A–C) Data shown for EVs isolated by differential centrifugation characterizing surface marker profiles and fractional size by NanoFCM in (A) heart, (B) kidney and (C) lung preparations obtained from enzymatic and ATD protocols. (D–F) SEC isolation of small EVs and subsequent characterization of surface marker profiles and fractional size in (D) heart, (E) kidney and (F) lung. (G) Detection of cardiac troponin+ small EVs in SEC samples. Data are mean ± SEM, *n* ≥ 3 per group. Unpaired *t*‐test; *p < 0.05, **p < 0.01, ***p < 0.001.

To understand the impact of different tissue dissociation approaches on the variability of EV preparations with respect to their physical characteristics and profiles, we next examined EV fractions from heart, kidney and lung preparations using both enzymatic and ATD methods. Using these two approaches and assessing EVs isolated by differential centrifugation, we could observe a large overlap of EV‐related parameters but also distinct and organ‐specific differences in mean EV size and marker‐based characterization of EV fractions. EVs were significantly larger in the enzymatic digests from the kidney, whereas size did not differ in lung and heart samples (Figure 4A–C). EV characterization indicated higher purity for ATD‐disintegrated samples with significantly higher levels of CD9+ EVs in lung samples (Figure 4C).

SEC isolated small EVs showed comparable trends across all organs with the exception of significantly larger particles recovered from enzymatically digested hearts (Figure 4D–F); moreover, significantly increased CD9+ particles were enumerated in kidney (Figure 4E) and lung ATD samples (Figure 4F). Cardiac troponin was not significantly different between ATD and enzymatic samples (Figure 4G). In contrast to EVs isolated by differential centrifugation, where no differences were observed with respect to the percentage of EVs positive for cell specific markers, some differences were observed in EVs isolated by SEC. Significantly more EVs were CD41+ in heart ATD samples and CD31+ in lung ATD samples than their respective enzymatically digested counterparts (Figure 4D,F).

In accordance with MISEV guidelines, we proceeded to assess markers of categories 1–5, meaning markers for protein‐content‐based EV characterization, including CD63 (category 1; similar to CD9 as a category 1 marker), flotillin (category 2), Apolipoprotein A1 (categories 3 and 5), and calnexin (category 4). Protein content was measured using a BCA assay with highest concentrations detectable in kidney samples and standardized prior to loading (Figure S8A,B). Complete blots of protein markers investigated for SEC purified EVs can be found in Figures S8C‐E and S9A. CD63 levels were highest in kidney samples with increased levels in ATD versus enzymatic samples (Figure S9B). ApoA1 was generally low in all samples with slightly higher levels in ATD processed organs (Figure S9C). Flotillin1 was detectable in ATD samples, whereas absence was noticeable in enzymatically digested organs (Figure S9D). Calnexin was only detectable in whole tissue heart lysates (Figure S9E), with multiple bands detectable. Calnexin is reported at different molecular weights by Western blot depending on utilized antibody and buffers/preparations. Indeed, we have observed both smears as well as multiple bands in heart lysates, with more than one band also detectable in cell extracts (Figure S10B). Different molecular weights for calnexin are reported in the literature, with a 150 kDa species reported in murine liver lysates (Gong et al. 2025) in contrast to an ∼90 kDa species detected in human liver slice lysates (Geng et al. 2025). In murine brain homogenates a 75 kDa species has been identified (Huang et al. 2020). These discrepancies may be due to different preparation/running conditions or differential post‐translational modification of calnexin (Paskevicius et al. 2023). Evaluation of CD63 expression and calnexin on EVs isolated by differential centrifugation demonstrated similarly low levels of calnexin expression and the presence of CD63 when comparing ATD and enzymatically processed kidney (Figure S10A,B).

### 3.4 Cell Viability Differs Between the Compared Tissue Disintegration Methods

Virtually all cells are prone to release EVs. The production of different EV subtypes varies according to the activation status of the cell and can be confounded by cellular damage (Tucher et al. 2018). To understand the impact of different organ‐inherent cell types as well as differences in cell viability and activation on variability of EV detection in whole organs, we performed flow cytometry analyses of the enzymatic and ATD processed organ samples from aliquots taken prior to EV isolation.

**FIGURE 5:**
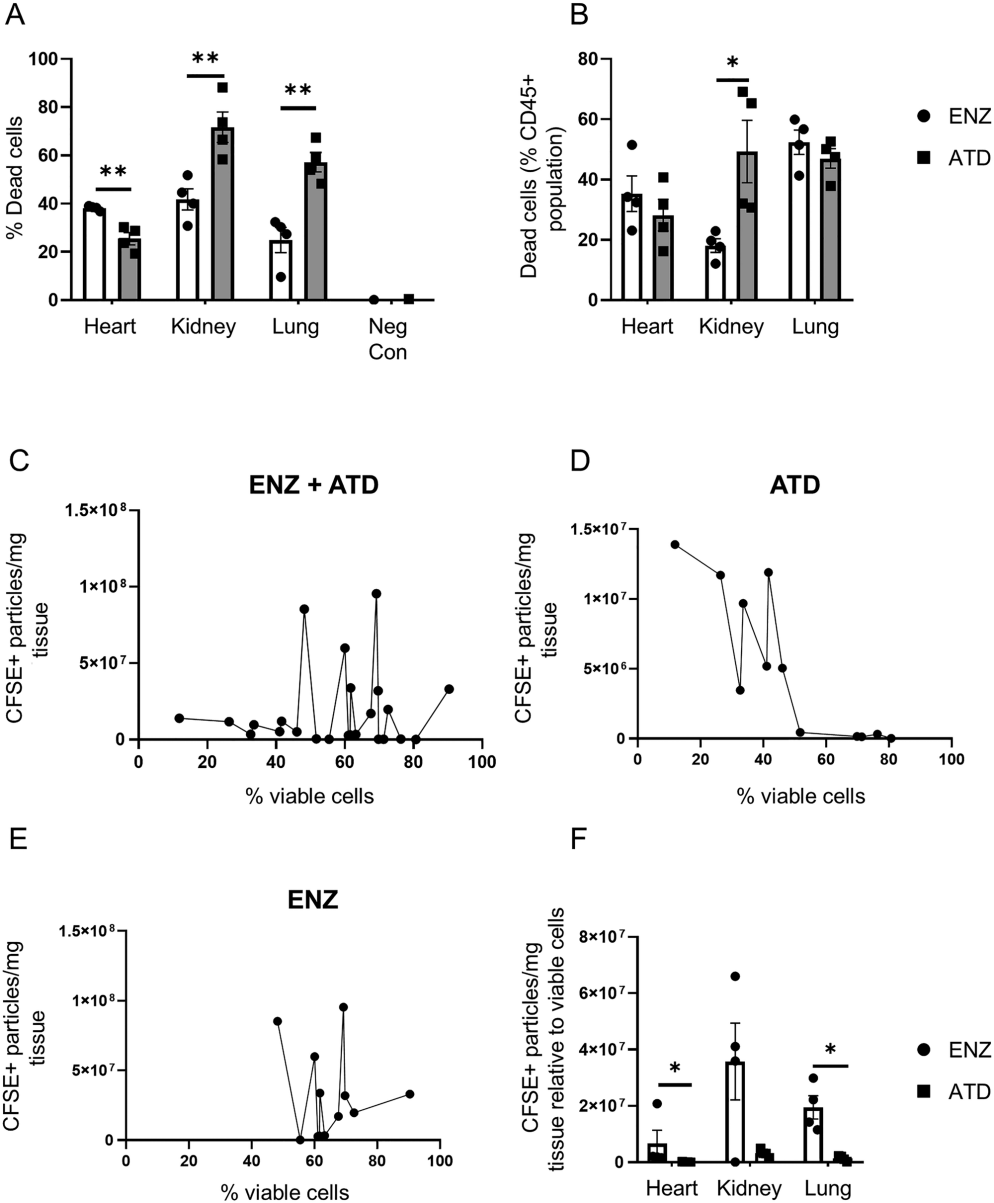
**Cellular viability differs using two distinct organ dissociation methods**. (A) Percentage dead cells in each organ processed by either enzymatic digestion or ATD as assessed by flow cytometry using Fixable Viability Dye eFluor780. (B) Dead cells in each organ expressed as percentage of CD45+ population. (C) Correlation analyses of both tissue processing methods, (D) ATD only or (E) enzymatically digested only samples across all organs performed using a Spearman's correlation test and plotted for x = % cell viability as assessed by flow cytometry and y = total CFSE+ particles/mg tissue. (F) CFSE+ particles per mg tissue relative to live cells. Data are mean ± SEM, *n* ≥ 3 per group. Unpaired *t*‐test; *p < 0.05, **p < 0.01.

For lung and kidney, we observed reduced cell viability following ATD compared to enzymatic digestion, whereas viability was higher for ATD heart samples (Figure 5A). Focusing on CD45+ cells, no significant differences in dead hematopoietic/leukocytic cells were notable in lung and heart samples, but an increased percentage of dead cells was observed for ATD kidney samples (Figure 5B). Viable CD45+ cell number was low overall, with lowest values quantified in hearts (Figure S10C). This is in accordance with the literature (Pinto et al. 2016). Correlation analyses between FACS‐based cellular viability (% viable cells out of total events) and differential centrifugation isolated CFSE+ particles per mg of tissue was performed to understand whether cellular stress/death induced by either tissue dissociation technique impacted the occurrence and detectability of EVs. No significant correlation for overall effects was observed when taking into consideration all samples (Figure 5C). Nonetheless, when focusing on the ATD‐processed samples, without organ‐based subgrouping, a Spearman correlation of *r* = −0.860 with *p* = 0.001 was observable (Figure 5D; Figure S10D shows integrated linear regression), whereas the enzymatic analyses did not show correlation between viability and particle yield (*r* = 0.147; *p* = 0.651) (Figure 5E). When calculating the relative amount of CFSE+ particles per mg tissue by multiplication with the % viable cells as assessed by flow cytometry, increased values were detectable in the enzymatically digested hearts and lungs compared to the ATD group (Figure 5F).

### 3.5 Platelet and Immune Cell Activation Differs Between Tissue Disintegration Methods

The ATD mechanical dissociation method entails application of shear and enzymatic approaches also include steps which require application of mechanical stress (trituration, cutting); additionally, the enzymes themselves can cleave relevant surface proteins. Since EV yields can not only be impacted by cell viability but also by cell reactivity, we next assessed the activation state of both platelets and leukocytes, as well as the formation of platelet‐leukocyte aggregates. In fact, platelets and leukocytes are prone to activation and release EVs when exposed to mechanical stress (Holme et al. 1997; Pieper et al. 2018; Radley et al. 2019; Sakariassen et al. 1998). A flow cytometry‐based approach was used following blood removal from all organs by exsanguination and flushing as described in the Methods section. Cellular activation was determined by quantifying (i) formation of platelet‐leukocyte aggregates, (ii) the ADAM17‐iRhom2‐based shedding of surface CD62L on leukocytes (Cappenberg et al. 2019), and (iii) surface mobilization of CD62P on platelets and iv) shift to a high affinity conformation of the GPIIb/IIIa integrin on platelets (Margraf, Nussbaum, et al. 2019).

#### 3.5.1 Heart

**FIGURE 6:**
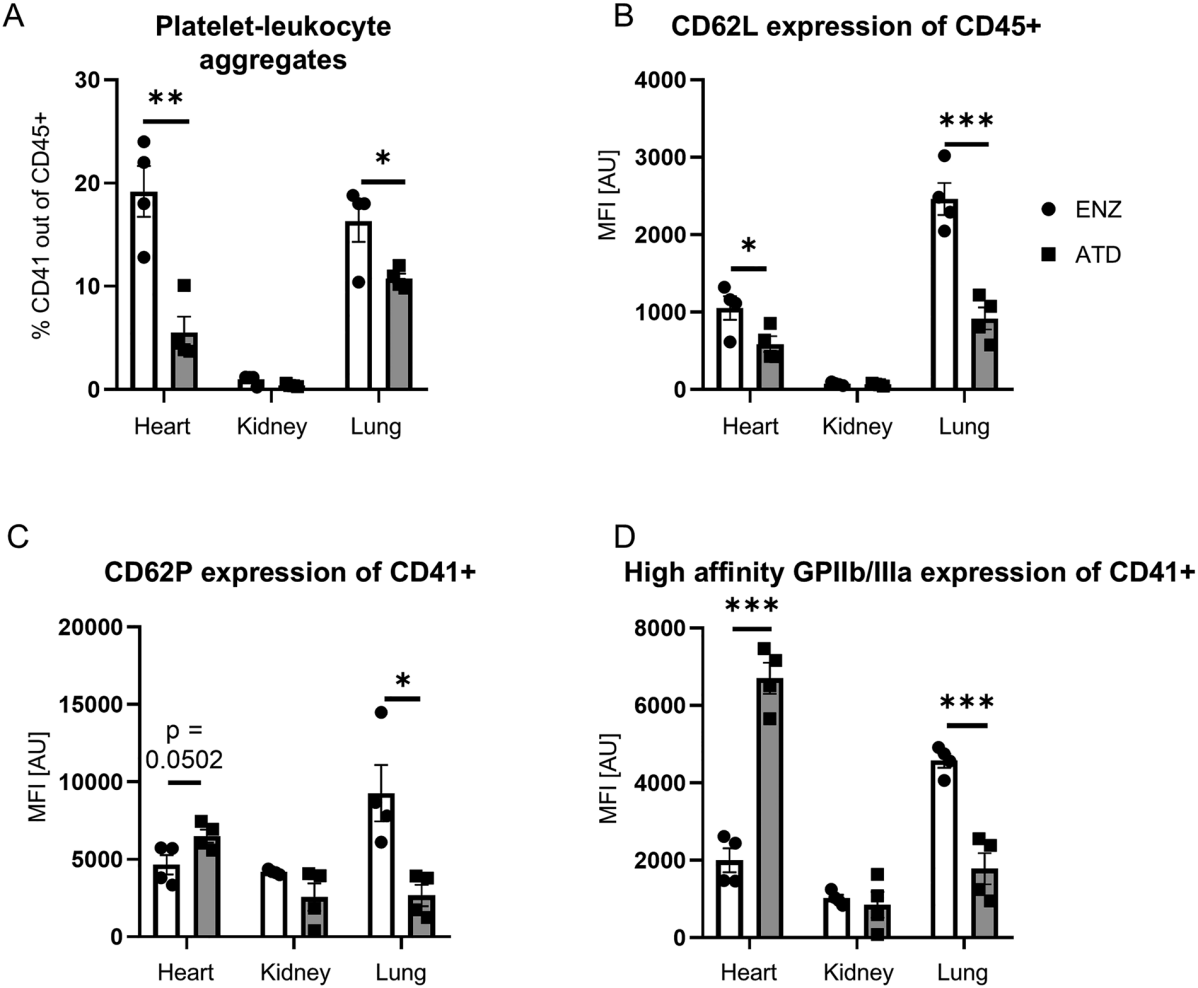
**Cell activation and aggregate formation in organ extracts**. (A) Percentage of platelet‐leukocyte aggregates out of CD45+ events in organs processed by enzymatic digestion or ATD. (B) CD62L surface levels on CD45+ cells from organs processed by enzymatic digestion or ATD. (C) Expression of platelet activation markers CD62P and (D) activated integrin GPIIb/IIIa on CD41+ platelets from organs processed by enzymatic digestion or ATD. Data are mean ± SEM, *n* ≥ 3 per group. Unpaired *t*‐test for each organ; *p < 0.05, **p < 0.01, ***p < 0.001.

Higher levels of CD41+ platelet‐CD45+ leukocyte aggregates could be observed in enzymatically digested heart samples (Figure 6A), potentially indicating either increased cellular activation or a reduced dissociation of aggregates due to shear when compared to the ATD methodology. A modest difference in platelet CD62P mobilization could be noted together with a significant increase in the high affinity conformation of GPIIb/IIIa in the ATD samples (Figure 6C,D). Similarly, CD62L shedding was increased in the ATD samples (Figure 6B), all indicative of increased cellular activation during the ATD process applied to generate the experimental samples.

#### 3.5.2 Kidney

Slightly, but non‐significantly, higher PLA levels in the enzymatically digested kidney samples could be observed (Figure 6A). No significant differences in platelet surface CD62P, high affinity GPIIb/IIIa levels nor CD62L shedding could be noted (Figure 6B–D).

#### 3.5.3 Lung

Within the lung samples, increased numbers of CD41+/CD45+ platelet‐leukocyte aggregates were quantified in the enzymatic digest group (Figure 6A). Thus, platelets within the enzymatic group showed increased CD62P surface expression, as well as an increased binding of the platelet‐specific reporter antibody directed against the high affinity conformation of the GPIIb/IIIa integrin (Figure 6C,D). When assessing leukocyte surface markers, higher CD62L expression was notable in the enzymatic versus ATD group within the lung, supporting the notion that the increased interaction of platelets and leukocytes may primarily be a platelet‐mediated effect in this organ and setting (Figure 6B).

## 4 Discussion

In this study, we utilized two distinct tissue processing methods to characterize EV isolation profiles from intact organs. We studied samples of intact hearts, kidneys, and lungs obtained using both a standardized enzymatic protocol, as well as a mechanical automated‐tissue‐dissociation method, which offers fast sample processing times. Overall, enzymatic digestion resulted in higher yields of EVs. Properties of EVs varied amongst organs with the largest EVs obtained from the kidney. EV phenotyping showed increased fractions of CD9+ particles in ATD‐processed lungs compared to enzymatic digest when EVs were isolated by either differential centrifugation or SEC. The highest Ter‐119 fraction was detected in lung and heart samples, indicating a degree of erythrocyte contamination, despite the blood‐removal step.

Previous work has shown that EVs can be prepared from sliced tissues (Crescitelli et al. 2021; Huang et al. 2020). Efficient EV isolation from different organs requires optimization of isolation strategies for each organ and comparative systematic assessment of dissociation protocols. To gain an unbiased overview of the isolated fractions and amounts of EVs, we chose a comparative strategy in which similar liberase‐based digestion was performed for each organ whereas the recommended organ‐adapted distributor‐optimized settings of the commercially available ATD were used for tissue dissociation and EV isolation. An ATD device for tissue dissociation and EV isolation appears a plausible approach considering its fast‐processing times, comparable cellular viability and yield, multimodal use of tissue samples and potential high costs of digestion enzymes (Soteriou et al. 2023). It has been proposed that a tissue homogenizer might lead to contamination of samples with intracellular vesicles (Crescitelli et al. 2021). Our data indicates that the EV yield was lower in the ATD‐treated samples compared to enzyme‐digested samples. Western blot analysis showed low detection of calnexin (exclusion marker for EVs as an endoplasmic reticulum protein) in both enzymatic and ATD processed EV samples, thus indicating reduced cross contamination with intracellular vesicles. Differences in flotillin levels are surprising but again potentially attributable to differences in isolated EV subsets based on organ disintegration methodology applied. CD63 levels were also variable between preparations, although differential expression between cell types has been reported in the literature (Cashikar and Hanson 2019). Nonetheless, detection of apolipoprotein A1 as another marker of EV purity was low amongst all groups.

Recently, Han et al. (Han et al. 2024) have compared enzyme‐based (perfusion vs. digestion of tissue fragments) dissociation methods with culturing tissue fragments in the absence of enzymes for profiling protein markers from lung tissue‐derived EVs (Han et al. 2024). Perfusion of blood was found to be of importance for reduction of serum EV proteins and lipoproteins and meshing (straining) of tissue improved EV yield. These findings support our methodology as we also perfused blood and strained tissues post‐digestion for all samples. Perfusion of enzymes into the lungs resulted in the highest EV yield and is not a method that we have utilized. However, as acknowledged by the Authors, this is technically challenging and not relevant for human biopsies. What both studies reveal are variations in cell viability, EV yield and protein expression across organs as well as in relation to the separation techniques applied.

One major concern for tissue‐based EV isolation strategies is pre‐activation of and damage to cells, potentially leading to false reporting of EV fractions. Ideally, isolation of EVs from intact organs or tissue sections should feature high cellular viability, high EV yield, reduced cell damage, low processing‐associated cell activation, limited cost, and quick turnover. As EV characterization from whole tissues will facilitate a better understanding of EVs as effectors/communicators for cellular crosstalk within certain compartments it is of interest to the field to determine the impact of different tissue dissociation methods on both cell and EV biology. To determine the impact of the dissociation techniques on cell integrity and activation, we quantified cell viability and cell activation by flow cytometry. The percentage of dead cells was comparable across all organs after enzymatic digestion; however, differences were detected in the ATD samples. Additionally, cell viability was higher in enzymatically‐digested samples, indicating that mechanical stress induced by the ATD might reduce cellular integrity. These observations are contrasting to a recent study which reported that both conventional digestion approaches (including enzymatic techniques) and an ATD approach resulted in high cellular viability (Soteriou et al. 2023). Notably, different digestion protocols, suspension media, processing protocols as well as viability detection protocols were used; all these variables have the potential to impact the quantification of viable cells. Soteriou et al. did not examine the heart, whereas for kidney and lung they applied a propidium iodide viability detection assay, observing a trend toward higher viability with the standard enzymatic dissociation approach compared to when tissues were dissociated with the ATD (Soteriou et al. 2023). The ATD setup allows precise protocol adaptations. These include use of different mesh sizes as well as adaptation of rotation speed, direction and time, all of which will impact the shear stress exerted onto the tissue. Understanding the optimal settings for ATD processing of tissue for EV isolation remains to be investigated in a tissue‐dependent manner. The current work does, however, highlight ATD mediated tissue processing as a rapid alternative to enzymatic digestion and provides an opportunity for tissue processing without ‘batch effect’ issues, which may result from different lots of reagents. Notably, the ATD may impact cell viability through the application of mechanical stress. Reduced cell viability can impact EV yields (e.g., apoptotic EVs (Kakarla et al. 2020)) and thus researchers must include adequate controls to confirm that the isolated EVs reflect the tissue configuration or biology rather than artificially induced stress reactions. Nonetheless, in the current study—with exception of the heart—enzymatic preparations showed increased cellular viability and yielded larger numbers of EVs compared to ATD preparations, which showed less viability and reduced EV counts, thereby pointing to non‐apoptosis related EV yield. Interestingly, when focusing on the activation of readily stimulated cells as “biosensors” for cellular activation, namely, platelets and leukocytes, our data indicate a more subtle outcome: presence of PLAs was higher in enzymatically‐treated than ATD‐exposed organs. PLAs indicate cellular, particularly platelet, activation (Jy et al. 1998; Margraf and Zarbock 2019), therefore this dataset suggests that enzymatic digestion may result in increased cellular activation compared to ATD. This finding correlates with the reduced CD62P and GPIIb/IIIa expression on platelets from lung samples prepared with ATD. Thus, tissue/cell specific responses to shear and enzyme cleavage may play a role.

The other possibility is that digestion methods result in dissociation of PLAs and that this occurs more readily with mechanical tissue disruption, although cell doublets can still be detected to varying degrees following ATD sample processing of different tissues (Soteriou et al. 2023). Increased levels of CD62L shedding in ATD heart and lung samples together with increased expression of the high affinity conformation of GPIIb/IIIa integrin on platelets in ATD heart samples, are testimony of significant cell activation within the respective tissues. Of note, CD62P surface mobilization and high affinity GPIIb/IIIa on platelets in the lung was lower in the ATD group, corroborating organ‐ and cellular subset‐specific differences in dissociation‐based pre‐activation. This appears plausible, as an ATD approach uses organ‐specified customized protocols, thereby implying a different degree of dissociation and impactful activation on tissues and cells. Interestingly, CD31+ EVs are a marker of both platelet and endothelial cell activation and we highlight that the amount of CD31+ EVs did not differ in heart and only in small EV preparations of the lung between enzyme‐ and ATD‐based dissociation even though platelet activation indices were significantly different in these organs. Thus, CD31 may not be a relevant marker of activation in this context. CD31+ EVs were higher in SEC isolated small EV populations, which correlates with literature indicating enrichment of adhesion proteins in small EVs (Jimenez et al. 2019).

Comparison of inter‐organ variation in the characterization of EV markers is another crucial component of the current study. Our analyses displayed a significant difference for the tetraspanin CD9 between kidney and lung. CD9 positive large EV levels were significantly higher in lung samples, potentially due to reduced loss of intact EVs, more successful tissue breakdown or reduced non‐EV contamination. This might be due to different anatomical structures and organ composition, such as reduced fibrous content of the lung and therefore easier mechanical accessibility. The levels of CD326 and CD31 positive particles were not impacted by cell dissociation method, except for SEC isolated EVs from the lungs, where significantly higher numbers of CD31+ EVs were found in the ATD preparation. The highest levels of CD326 and CD31 positive particles were observed in heart and kidney samples. These markers result from activation, cellular composition and partially recruitment, since CD31 can derive from endothelial cells as well as platelets, which are known to be of utmost relevance for mediating inflammatory cell recruitment in the kidney (Margraf, Ley, et al. 2019). The mechanism behind the higher detection of these EV markers in our samples remains unclear. Preponderance of specific cellular subsets, as well as impact of digestion approaches, including the variation in incubation time, penetration and success of organ decomposition based on enzymes could be one potential factor, in addition to organ‐specific susceptibility for cell‐activation and damage‐sensing.

Inclusion of Ter‐119 staining showed low levels of blood contamination in our samples indicating successful flushing of organs prior to harvest, thus we anticipate that contamination from blood cell EV populations will be minimal. Indeed, while platelets can be rapidly activated, levels of CD41+ EVs were low across all organs and conditions. Nonetheless, we are aware that differential ultracentrifugation may risk generation of aggregates in EV isolates. Since NTA approaches can be imprecise, we focused on NanoFCM‐based labelling strategies, size cutoff and application of additional SEC‐based approaches for EV isolation and identification. With regards to centrifugation steps applied for the isolation of EVs, our protocol included a 13,000 × *g* step to pellet apoptotic bodies. It is worth noting that such a step may also pellet fractions of very large EVs. Nonetheless, this method has been used successfully (Oggero et al. 2021) and was equally applied across all conditions, thus any bias introduced would be similar across all samples.

In addition to protocols for EV isolation from whole tissues and the detection of organ‐based EV differences, the current study highlights differences and limitations for EV detection. Previous studies have demonstrated that high levels of non‐EV particulate contaminants are of concern as they distort methods of quantification. For example, high concentrations of lipoproteins can give false positive events on Nanoparticle Tracking Analysis platforms including the Nanosight (Takov et al. 2017). Such an outcome can occur in the absence of fluorescence, since NTA can be applied to investigate protein aggregates in the study of Alzheimer's (Lu and Murphy 2018). Several different techniques for EV determination were used in this study, including NanoFCM, super resolution microscopy, electron microscopy, Western blot and NTA. Each technique features distinct benefits but also carries limitations. As an example, it is important to be aware of the non‐specific nature of NTA‐based size determination of EVs. For this reason, we utilized the NanoFCM, in which fluorescent labelling aids in adequate analysis of EVs. The use of BODIPY or CFSE to label EVs, as performed in our assays, relies on binding to thiols and amines, respectively, which may result in the recording of false positive events due to protein aggregates, membrane debris containing transmembrane or associated proteins or lipoprotein contamination. Nonetheless, previous studies have applied BODIPY or CFSE to detect EVs (Fortunato et al., 2021; Headland et al. 2014; Pospichalova et al., 2015; Oggero et al. 2021). To validate staining of EVs with CFSE, we have used triton ablation for differentially centrifuged EVs, as previously reported (data not shown) (Brealey et al., 2024; Hussain et al., 2024), which significantly reduced particle number indicating that the positive signal we observed was genuinely due to membrane‐bound particles. In these analyses, we found CFSE to be unsuitable for labelling SEC‐isolated EVs, and this may be due to the enrichment of specific populations of EVs, which do not contain sufficient esterases to achieve fluorescence activation. It is known that EVs isolated from some cell types are not stained with CFSE (Tertel et al., 2022). Studies indicate that CFSE does not produce artifactual dye aggregates when compared to lipid binding dyes, although background fluorescence can be problematic if not adequately diluted out or removed prior to analysis (Morales‐Kastresana et al., 2017; Tertel et al., 2022). The addition of control tests where only the dye was added, routinely used in all of our analyses, typically yielded negligible signal indicating that at the dye concentrations used, dye aggregates are unlikely to be a source of significant false positive events. We have previously used BODIPY to label EVs (Headland et al. 2014; Oggero et al. 2021) when detecting EVs by Imagestream analysis. Whilst BODIPY is more likely than CFSE to stain lipoproteins in the experimental samples, the use of SEC would guarantee a lower lipoprotein contamination, a finding confirmed by Western blot. The latter, to detect presence of Tetraspanin, is a reliable method to validate the genuine presence of EVs. Importantly, flow cytometric determination of cellular viability revealed a rather high percentage of dead cells in samples prepared with either protocol and across all organs. Therefore, it cannot be excluded that parts of EV isolates might derive from dead cells; however, apoptotic body removal was performed and EV yields were greatest from enzymatic dissociation of tissues, which also resulted in higher cellular viability than processing by ATD.

Characterization of EVs relies on the application of different techniques. In the current study, we have included imaging approaches (Cryo‐TEM and super resolution microscopy; ONI). Both techniques feature distinct benefits and limitations. Nonetheless, both offer the opportunity to examine the variety and diversity of examined samples. Whilst these techniques were not performed uniformly on all samples, both provide evidence of EV isolation and validate the isolation procedures used herein for recovering EVs from whole tissues. Future studies are required to examine differences in EV structure that may be uncovered through comparative analysis of these techniques (Xu et al. 2024).

A potential limitation of this study is the use of KRN mice expressing a transgenic T‐cell receptor for glucose‐6‐phosphate isomerase peptide. While the effect of the transgene on organ‐specific EV production is unknown we think it is of negligible relevance here since the same animal organs were used in both methods. Furthermore, flow cytometric assessment of cardiac tissue was performed according to previously applied techniques using a BD Fortessa instrument and after passing samples through a 70‐µm filter. Due to size limitations in detectors, standard flow cytometry does not allow detection of cardiomyocytes and thus assessment of their viability, which may result in an underappreciation of the contribution of these cells to the EV populations we studied.

To summarize, EVs have been successfully isolated from fresh, as well as post‐mortem tissues, from multiple species, using a range of isolation methods including ultracentrifugation, size exclusion columns and sucrose density gradient centrifugation (Huang et al. 2020; Konoshenko et al. 2018; Liangsupree et al. 2021). These techniques vary considerably in terms of yield, purity and processing times and have revealed species differences which may reflect differences in tissue fragility (Huang et al. 2020). In this study we have compared an enzymatic protocol with a novel ATD device. Enzymatic digestion has successfully released inter‐cellular EVs from tissues, with greater yields obtained than from tissue disruption alone (Crescitelli et al. 2020), a conclusion that our data also support. In addition, we provide information on purity of tissue‐derived EVs in relation to isolation procedures, data we could obtain through using the NanoFCM to identify EV populations rather than, for example, Western blot, which does not allow analysis at the level of individual particles (Bachurski et al. 2019; Liu, Tian, et al. 2022).

Utilizing two established techniques of organ disintegration followed by approved multimodal control of EV assessment, we show that both enzymatic digestion and a commercially available ATD technique allows collection of EV fractions but with significantly differing properties (Table 4). This can form the basis for establishing optimal protocols to be applied for biomarker profiling or to determine the functional properties of EVs in future investigations. In any case, this work showcases that isolation of intact EVs with differing properties is possible from intact organs, opening a new avenue for research endeavours for diagnostic as well as translational therapeutic approaches.

**TABLE 4:** **Comparison of tissue dissociation techniques. (Grading of + (more) to +++ (max))** Created in BioRender. Cooper, D. (2025) https://BioRender.com/la4qez3.

	Processing cost	Processing time	Cell viability	Cell activation	EV yield	Comments
ATD	+ (excluding equipment purchase)	+	+++ heart kidney lung	+ heart kidney + lung	+ heart ++ kidney + lung	Easy, fast processing but lower EV yield irrespective of isolation technique. Tissue dependent.
Enzymatic	+++	+++	++ heart + kidney +++ lung	+ heart kidney + lung	++ heart +++ kidney ++ lung	Increased processing time but higher EV yield irrespective of isolation technique. Better for cell analysis. Tissue dependent.

## Author Contributions

**Mohammed Tayab Hussain**: Data curation (equal); formal analysis (equal); investigation (equal); methodology (equal); validation (equal); visualization (equal); writing ‐ original draft (equal); writing ‐ review and editing (equal). **Shani Austin‐Williams**: Conceptualization (equal); data curation (equal); formal analysis (equal); investigation (equal); methodology (equal); validation (equal); visualization (equal); writing ‐ original draft (equal); writing ‐ review and editing (supporting). **Joel McCay**: Formal analysis (supporting); investigation (supporting); visualization (supporting). **Hedayatullah Hayat**: Investigation (supporting); methodology (supporting). **Thomas D. Wright**: Data curation (supporting); investigation (supporting); methodology (supporting). **Marilena Christoforou**: Data curation (supporting); investigation (supporting); methodology (supporting). **Ella Ykema**: Data curation (supporting); investigation (supporting); methodology (supporting). **Mauro Perretti**: Funding acquisition (equal); resources (equal); software (equal); writing ‐ review and editing (equal). **Dianne Cooper**: Conceptualization (lead); data curation (lead); formal analysis (equal); funding acquisition (equal); investigation (equal); methodology (equal); project administration (lead); resources (lead); software (equal); supervision (lead); validation (equal); visualization (lead); writing ‐ original draft (lead); writing ‐ review and editing (lead). **Andreas Margraf**: Conceptualization (lead); data curation (lead); formal analysis (equal); funding acquisition (equal); investigation (lead); methodology (equal); project administration (lead); software (equal); supervision (lead); validation (equal); visualization (lead); writing ‐ original draft (lead); writing ‐ review and editing (lead).

## Conflicts of Interest

The authors declare no conflicts of interest.

## Supporting information




**Supplemental Figure 1**: Representative NanoFCM profiles of EVs isolated by differential centrifugation from hearts processed by either enzymatic digestion or ATD.
Samples were analysed using a NanoFCM flow cytometer. Exemplary plots of size vs. concentration distribution (upper) and CFSE‐FITC vs. sidescatter‐area (lower) are shown.
**Supplemental Figure 2**: Representative NanoFCM profiles of EVs isolated by differential centrifugation from lungs processed by either enzymatic digestion or ATD. Samples were analysed using a NanoFCM flow cytometer. Exemplary plots of size vs. concentration distribution (upper) and CFSE‐FITC vs. sidescatter‐area (lower) are shown.
**Supplemental Figure 3**: Representative NanoFCM profiles of EVs isolated by differential centrifugation from kidneys processed by either enzymatic digestion or ATD.
Samples were analysed using a NanoFCM flow cytometer. Exemplary plots of size vs. concentration distribution (upper) and CFSE‐FITC vs. sidescatter‐area (lower) are shown.
**Supplemental Figure 4**: Antibody only and dye only controls show detection of negligible positive particles by NanoFCM.
(A) Controls were performed by addition of antibodies alone to sterile filtered PBS (upper) in comparison to actual EV (kidney)‐based signals (lower). (B) Addition of either CFSE or BODIPY alone to sterile filtered PBS (upper)in comparison to EV (kidney)‐based signals (lower).
**Supplemental Figure 5**: Exemplary flow cytometry gating strategy.
**Supplemental Figure 6**: Particle yield quantified by NanoSight NTA and purity assessment of EVs from enzymatically digested organs.
Isolated EVs were assessed by NanoSight for total particle yield (A). Purity of enzymatically digested tissue samples was assessed by NanoFCM for(B) EVs isolated by differential centrifugation and (C) EVs isolated by SEC. Purity was calculated as percentage of positively labelled particles (CFSE/BODIPY) out of total detected particles.
**Supplemental Figure 7**: Particle yield, purity and super resolution microscopy of EVs isolated from organs processed by either enzymatic digestion or ATD.
(A) EVs isolated by differential centrifugation were assessed by NanoSight for total particle yield and (B) percentage purity of EVs isolated by differential centrifugation calculated as percentage of positively (CFSE) labelled particles out of total detected particles by NanoFCM. (C) Percentage purity of EVs isolated by SEC calculated as percentage of positively (BODIPY) labelled particles out of total detected particles by NanoFCM. (D) Oni super resolution‐based detection of SEC isolated EVs. Enzymatic degradation across all organs showed a higher number of clusters than ATD‐isolation which stained positive for pan membrane markers. (E) Comparison of degradation methods by organ (standard error based on technical replicates) and (F) cluster length.
**Supplemental Figure 8**: Protein concentration and Western blotting of SEC isolated EVs.
(A) Protein concentration determined by BCA assay and (B) staining for total protein *via* TotalStain Q (Azure Biosytems). Complete Western blots for EV validation from SEC purified samples for (C) Flotillin, (D) ApoA1, (E) Calnexin and (F) Calnexin in whole heart lysate. Each blot is labelled as follows: the first letter denotes the tissue of origin: K‐ Kidney, H‐heart or L‐Lung. Following the first letter are either E for enzymatic or ATD and the number represents the biological replicate.
**Supplemental Figure 9**: Western blotting of SEC isolated EVs and quantification of protein expression from SEC isolated EVs. Complete Western blots for EV validation from SEC purified samples for (A) CD63 levels in enzymatic digests vs. ATD processed samples isolated via SEC. Each blot is labelled as follows: the first letter denotes the tissue of origin: K‐ Kidney, H‐heart or L‐Lung. Following the first letter are either E for enzymatic or ATD and the number represents the biological replicate. (B‐E) Densitometric quantification was performed using the Fiji ImageJ software integrated gel analysis tool. Data are mean ± SEM, n = 3 per group.
**Supplemental Figure 10**: Representative Western blots validating EVs purified by differential centrifugation.
Complete Western blots for EV validation from EVs isolated by differential centrifugation for (A) CD63 and (B) Calnexin. Each blot is labelled as follows: the first letter denotes the tissue of origin: K‐ Kidney, H‐heart or L‐Lung. Following the first letter are either E for enzymatic or ATD and the number represents the biological replicate. C28/I2 are a human chondrocyte cell line included for control purposes only. A and B refer to two distinct lysate preparations. C, D: Assessment of cell viability by flow cytometry following tissue disruption by either enzymatic digestion or ATD. (C) CD45+ cells out of total events. (D) Correlation analysis with indicated trajectory using a linear regression model across all tissues.
**Supplemental Table 1**: Weights of the organs used in the study.

## Data Availability

All relevant data are included in the manuscript and the supplemental material. Any additional information is available from the corresponding authors upon reasonable request.
