# Supplementary material for: Comparative Assessment of Whole Organ Tissue Processing Methods for the Isolation of Extracellular Vesicles From Intact Organs
Source: J Extracell Vesicles. 2025 Sep 3;14(9):e70127. doi: 10.1002/jev2.70127 (PMC12408365; doi:10.1002/jev2.70127)
Supplement: Supplementary file 1 — Supplemental Figure 1: Representative NanoFCM profiles of EVs isolated by differential centrifugation from hearts processed by either enzymatic digestion or ATD. Samples were analysed using a NanoFCM flow cytometer. Exemplary plots of size vs. concentration distribution (upper) and CFSE‐FITC vs. sidescatter‐area (lower) are shown. Supplemental Figure 2: Representative NanoFCM profiles of EVs isolated by differential centrifugation from lungs processed by either enzymatic digestion or ATD. Samples were analysed using a NanoFCM flow cytometer. Exemplary plots of size vs. concentration distribution (upper) and CFSE‐FITC vs. sidescatter‐area (lower) are shown. Supplemental Figure 3: Representative NanoFCM profiles of EVs isolated by differential centrifugation from kidneys processed by either enzymatic digestion or ATD. Samples were analysed using a NanoFCM flow cytometer. Exemplary plots of size vs. concentration distribution (upper) and CFSE‐FITC vs. sidescatter‐area (lower) are shown. Supplemental Figure 4: Antibody only and dye only controls show detection of negligible positive particles by NanoFCM. (A) Controls were performed by addition of antibodies alone to sterile filtered PBS (upper) in comparison to actual EV (kidney)‐based signals (lower). (B) Addition of either CFSE or BODIPY alone to sterile filtered PBS (upper)in comparison to EV (kidney)‐based signals (lower). Supplemental Figure 5: Exemplary flow cytometry gating strategy. Supplemental Figure 6: Particle yield quantified by NanoSight NTA and purity assessment of EVs from enzymatically digested organs. Isolated EVs were assessed by NanoSight for total particle yield (A). Purity of enzymatically digested tissue samples was assessed by NanoFCM for(B) EVs isolated by differential centrifugation and (C) EVs isolated by SEC. Purity was calculated as percentage of positively labelled particles (CFSE/BODIPY) out of total detected particles. Supplemental Figure 7: Particle yield, purity and super resolution mic [file JEV2-14-e70127-s001.pdf]

Supplemental Figure 1

EVs isolated by differential centrifugation

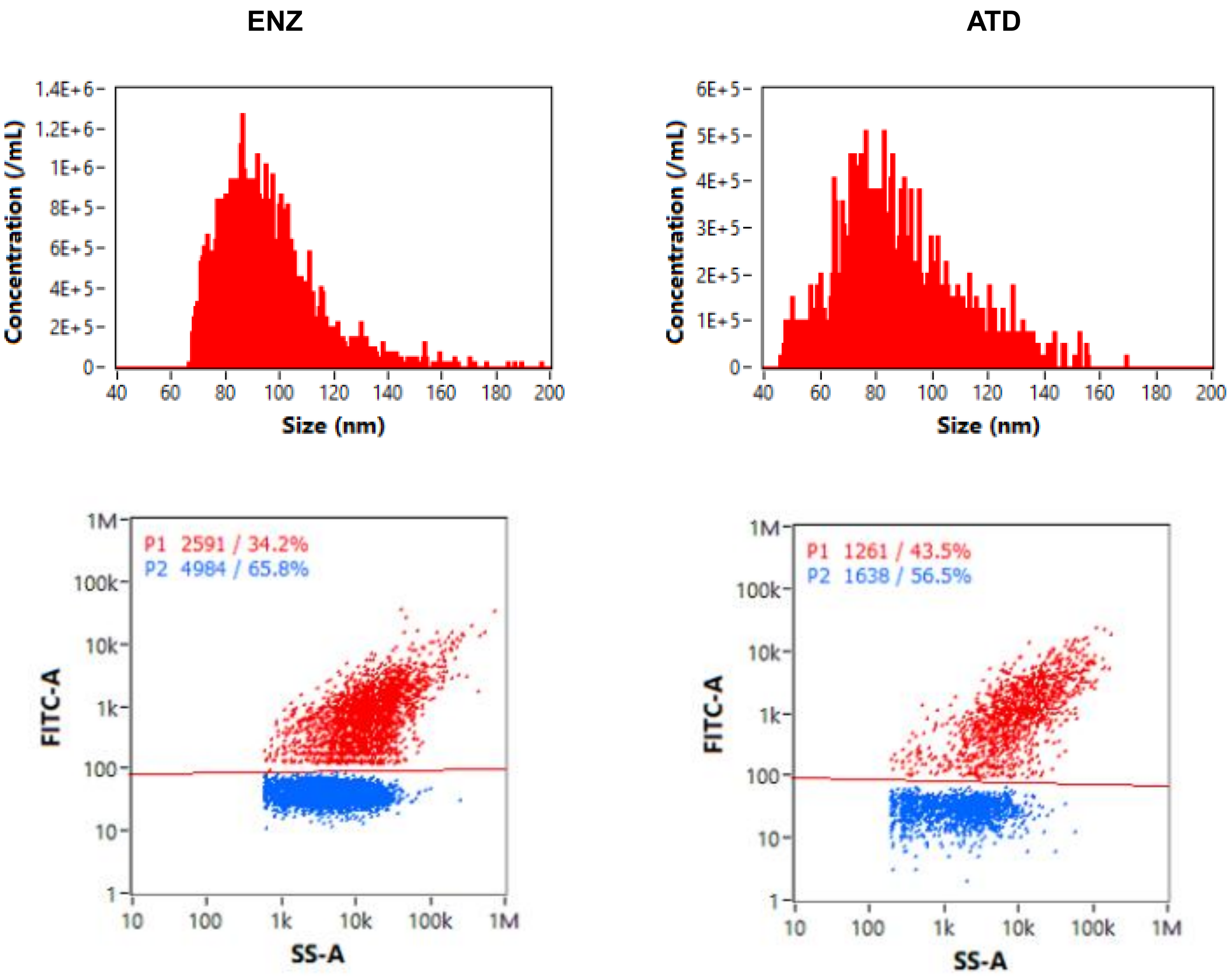

Supplemental Figure 2

EVs isolated by differential centrifugation

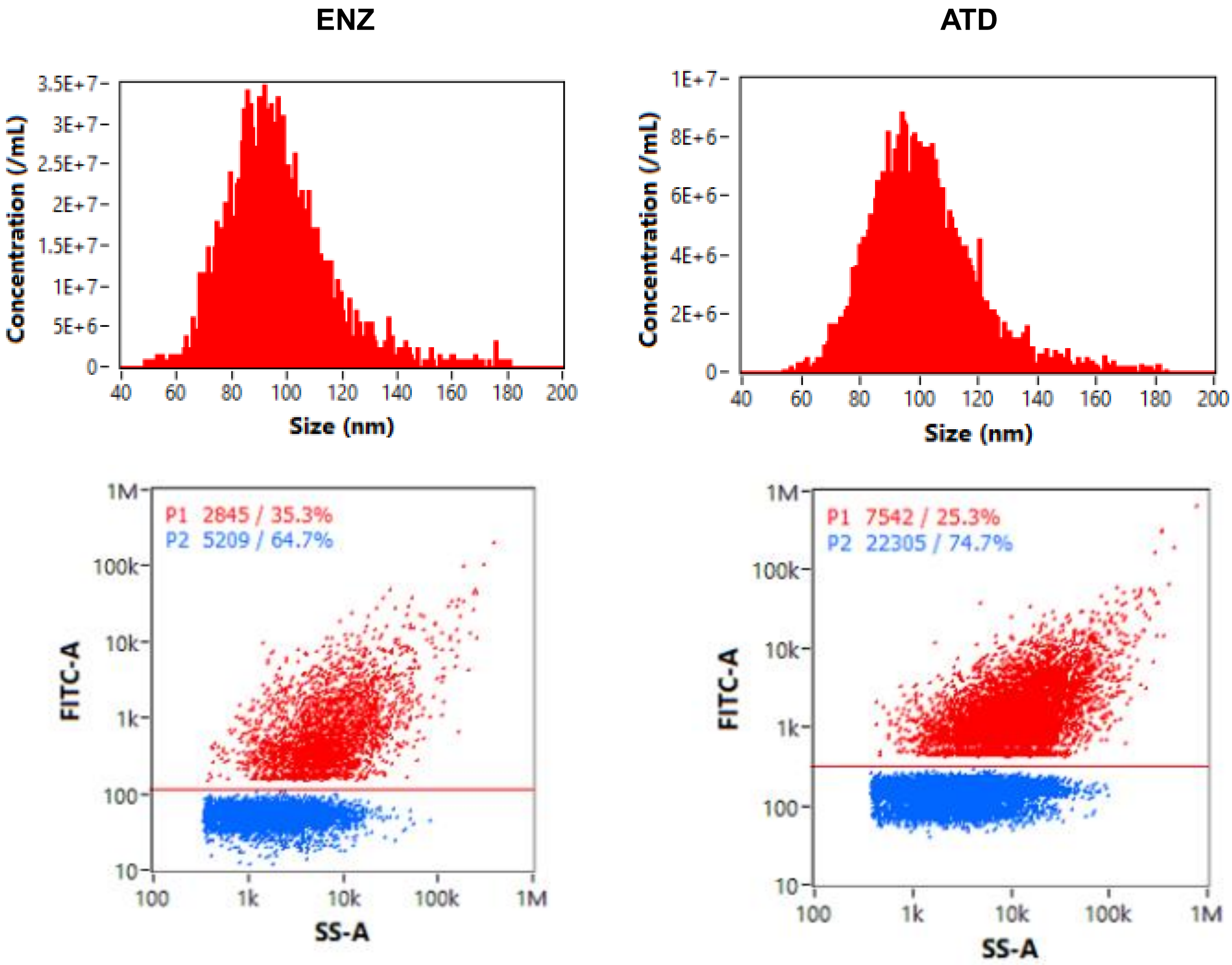

Supplemental Figure 3

EVs isolated by differential centrifugation

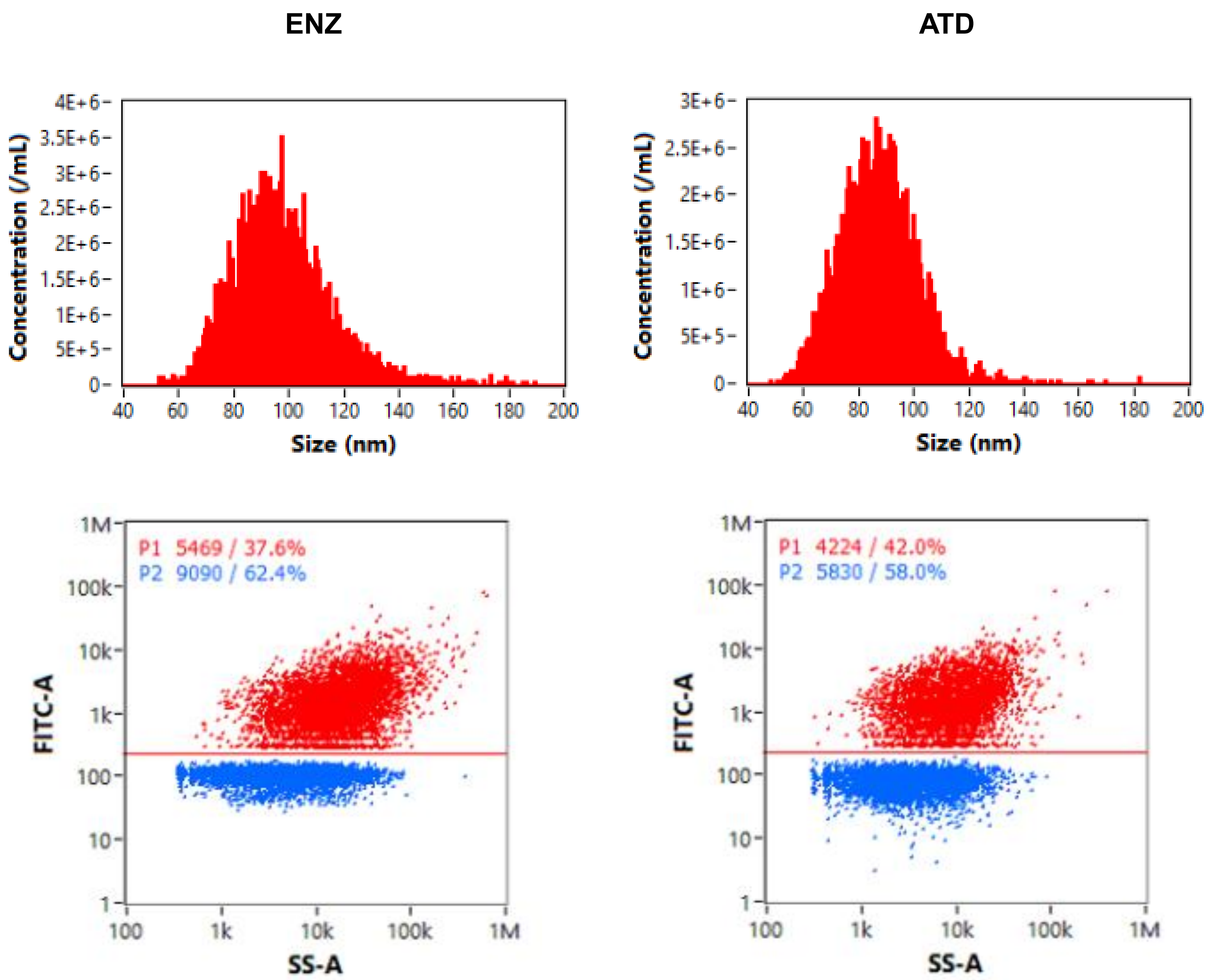

Supplemental Figure 4

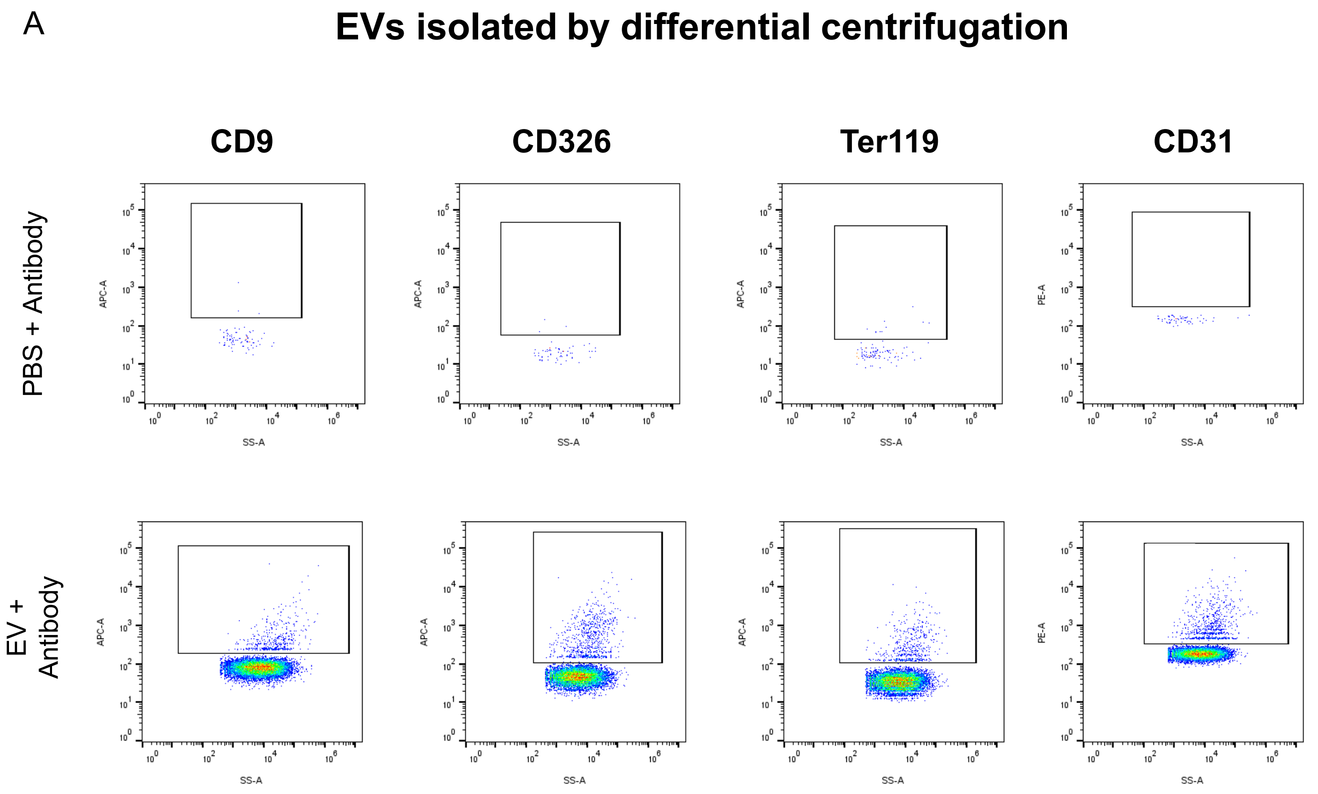

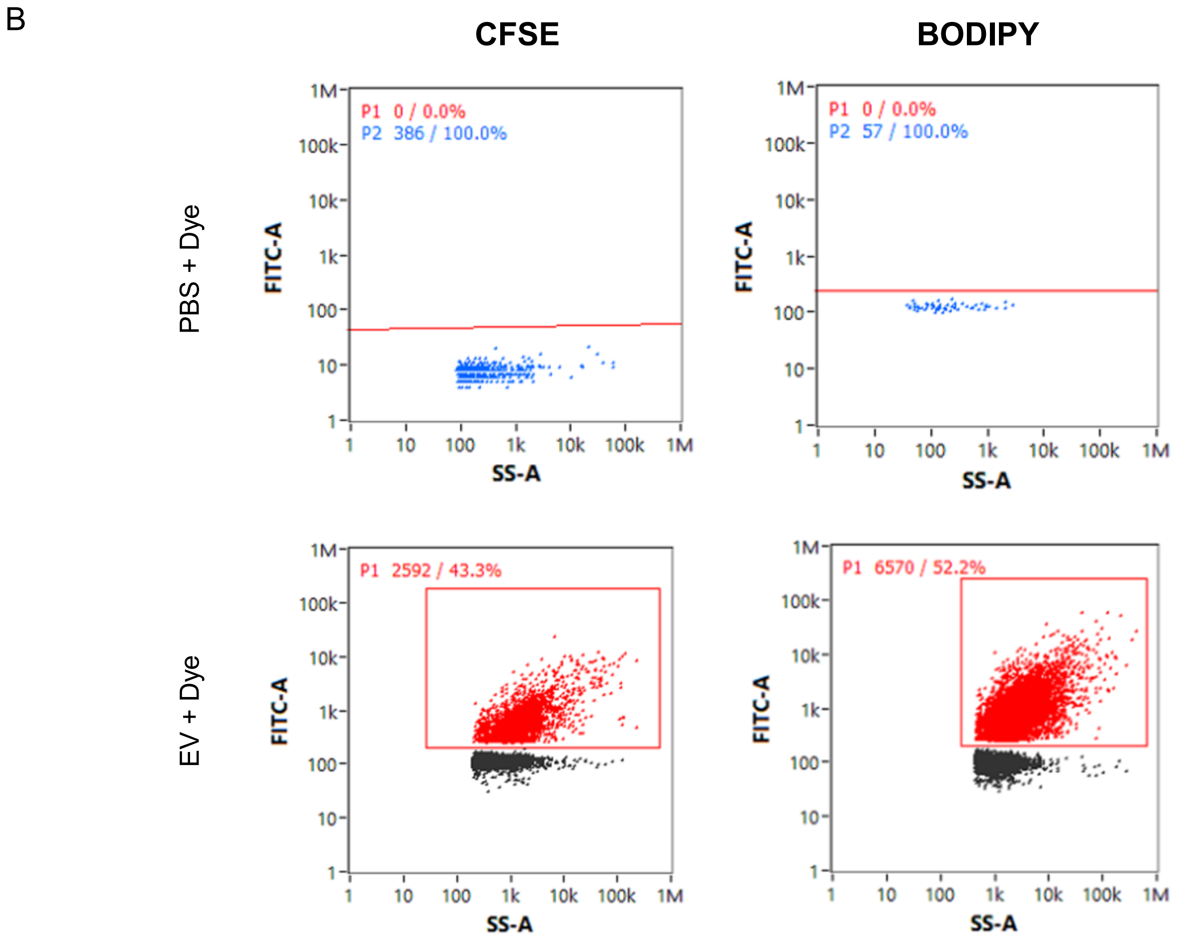

Supplemental Figure 5

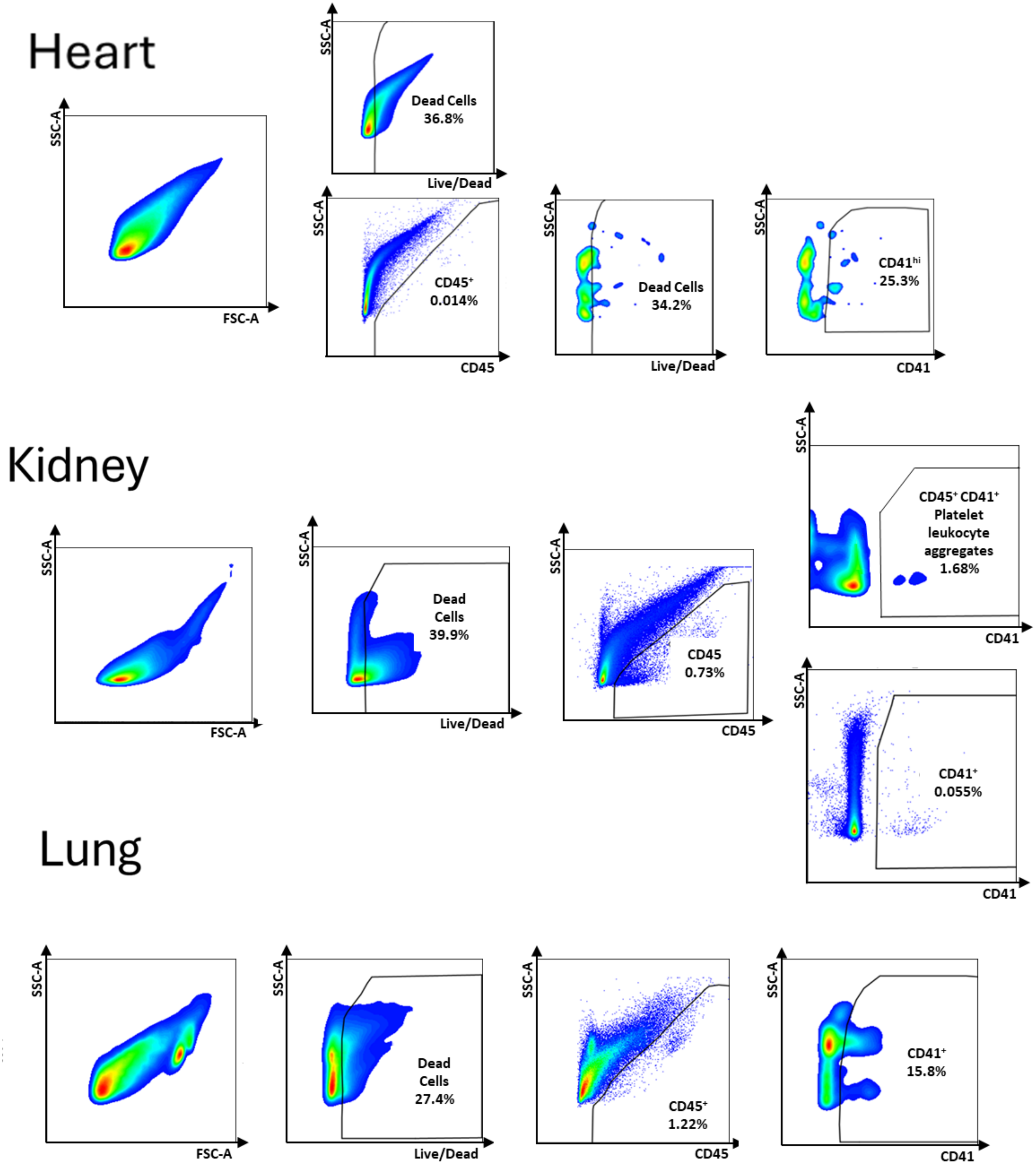

Supplemental Figure 6

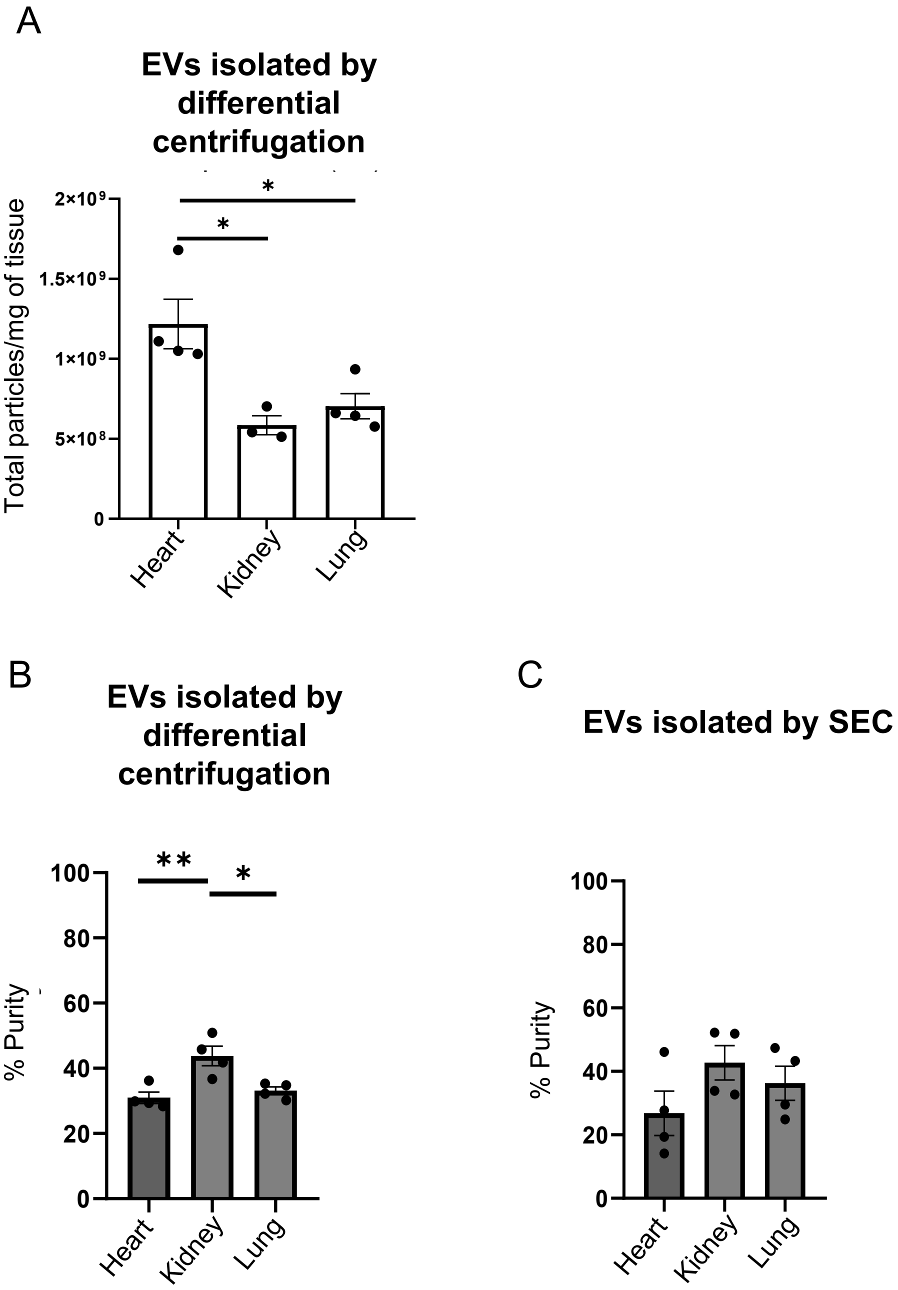

Supplemental Figure 7

EVs isolated by differential centrifugation

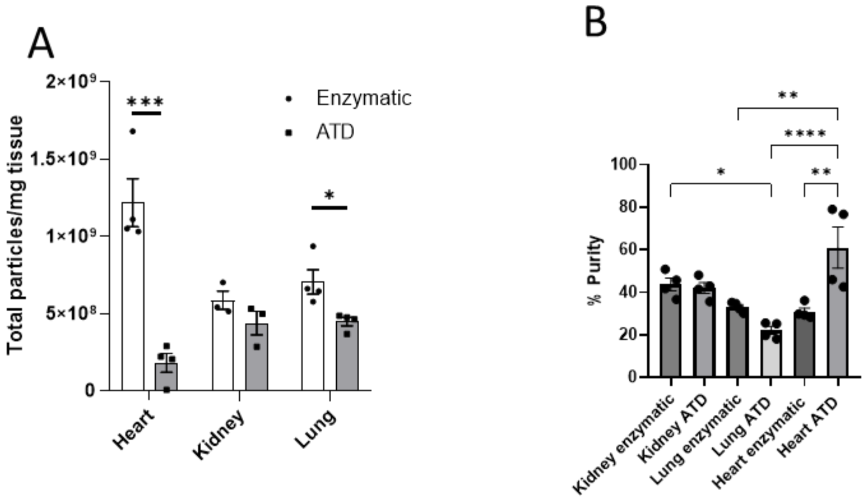

EVs isolated by SEC

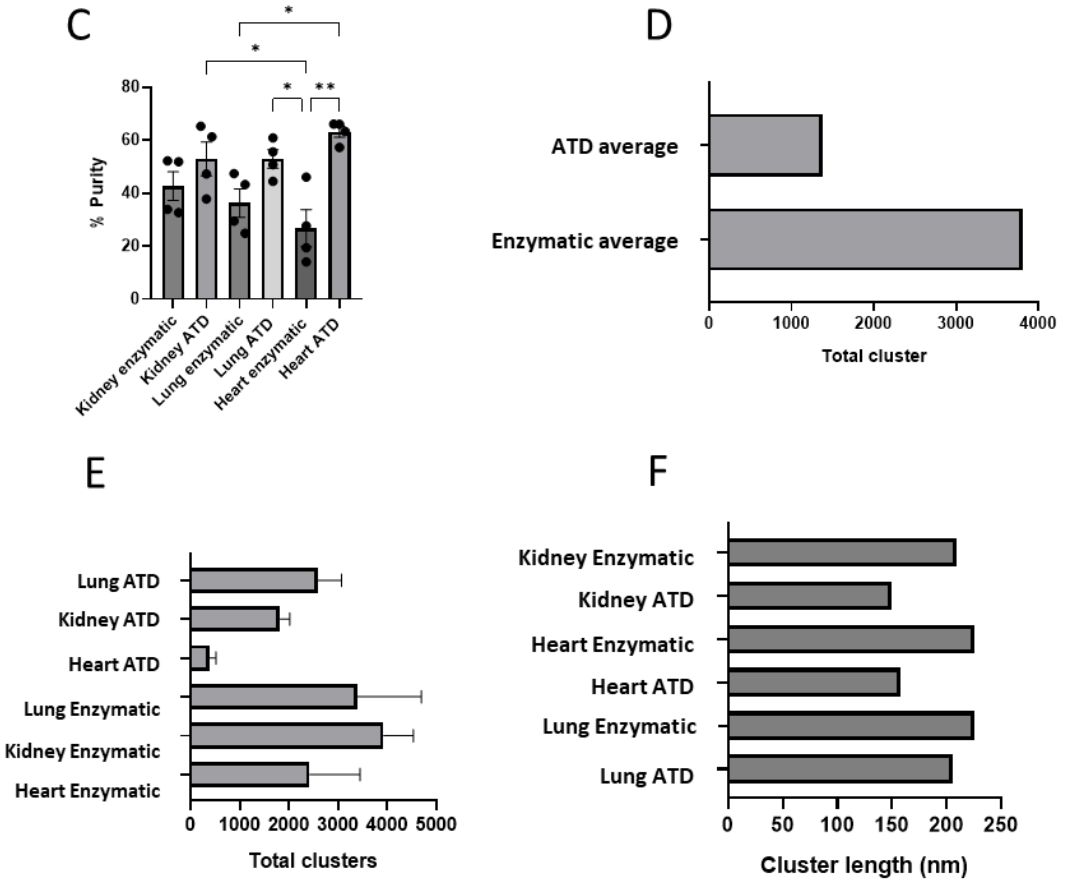

Supplemental Figure 8

EVs isolated by SEC

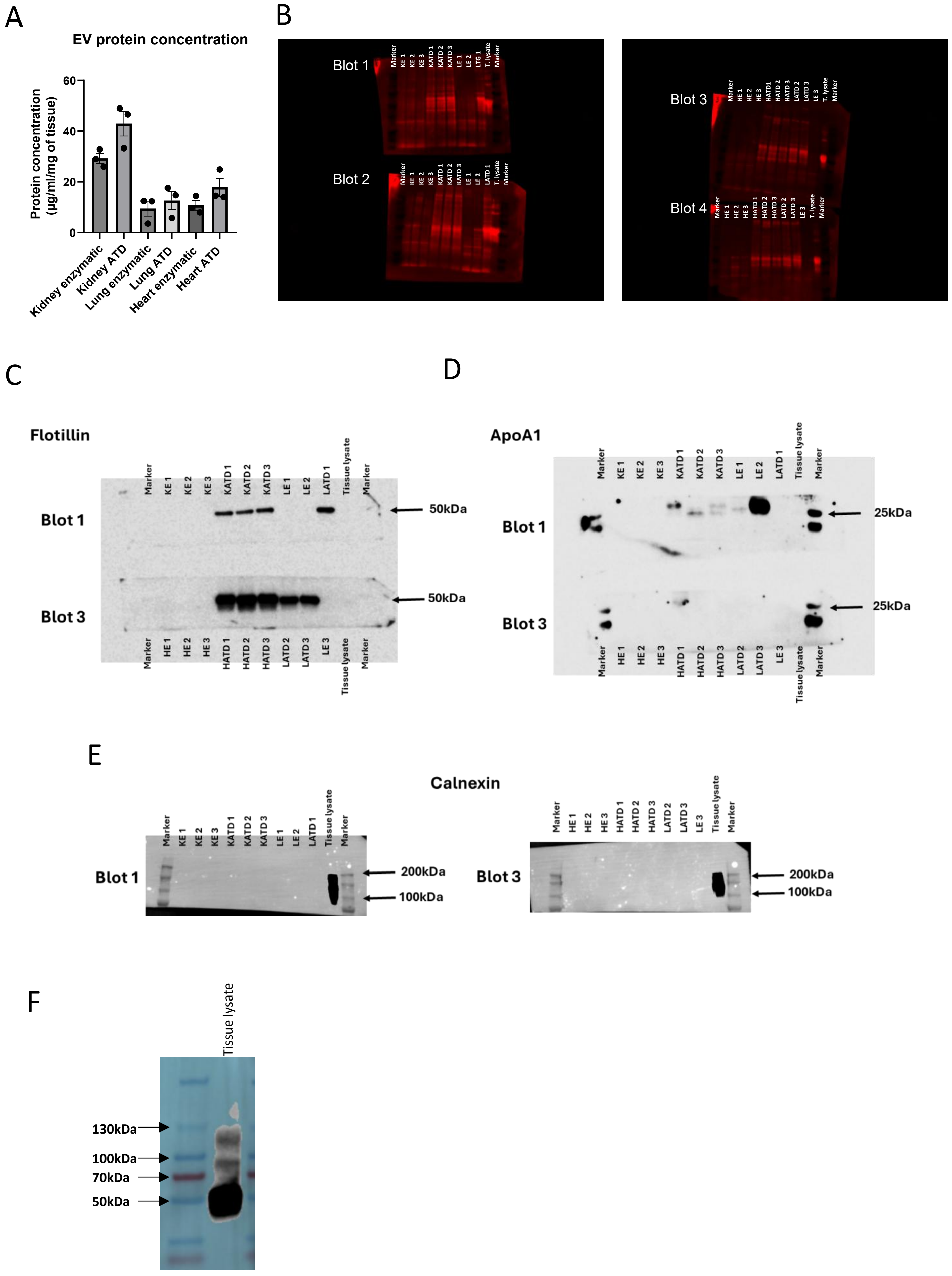

Supplemental Figure 9

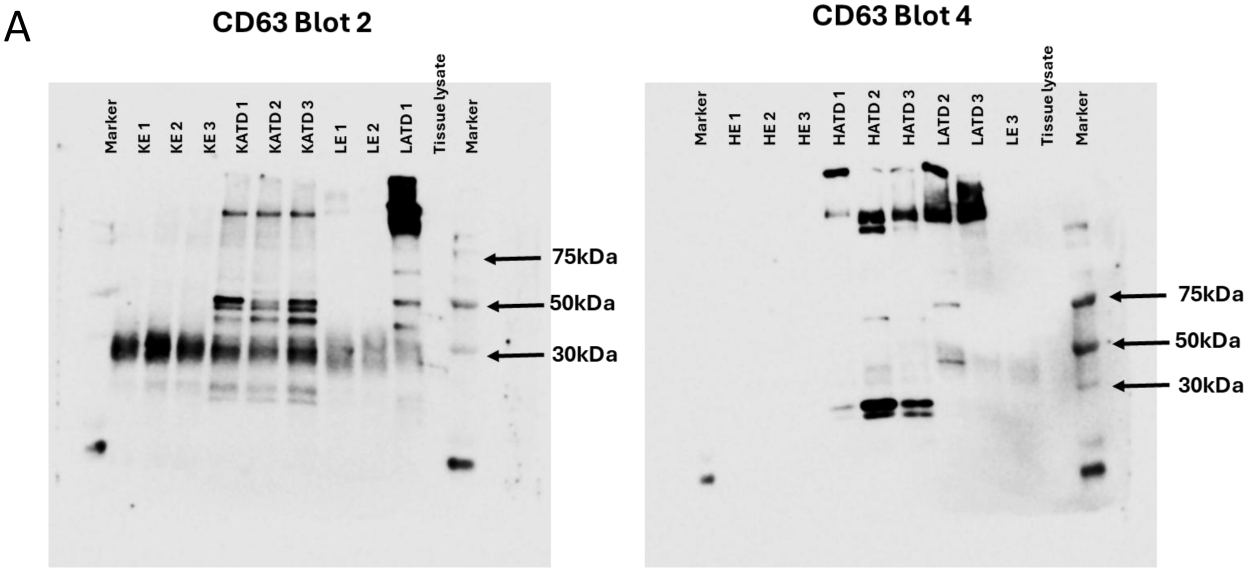

EVs isolated by SEC

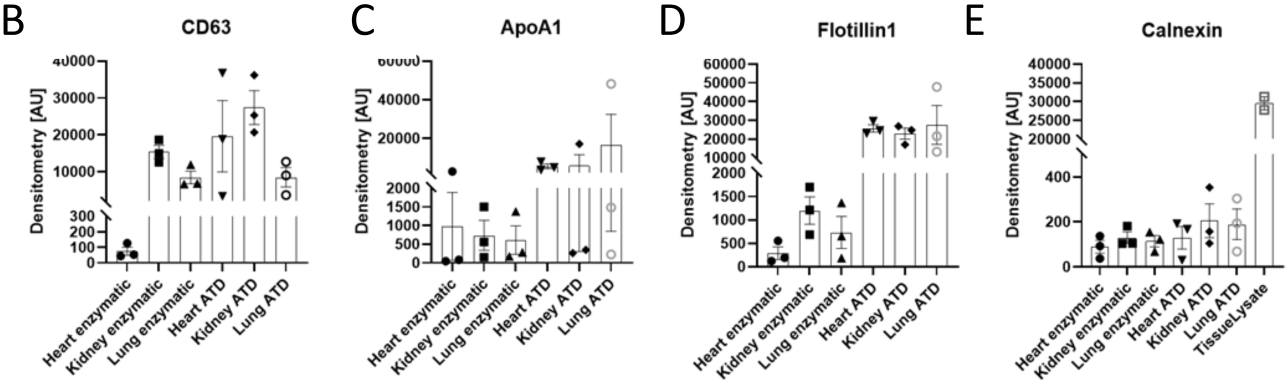

Supplemental Figure 10

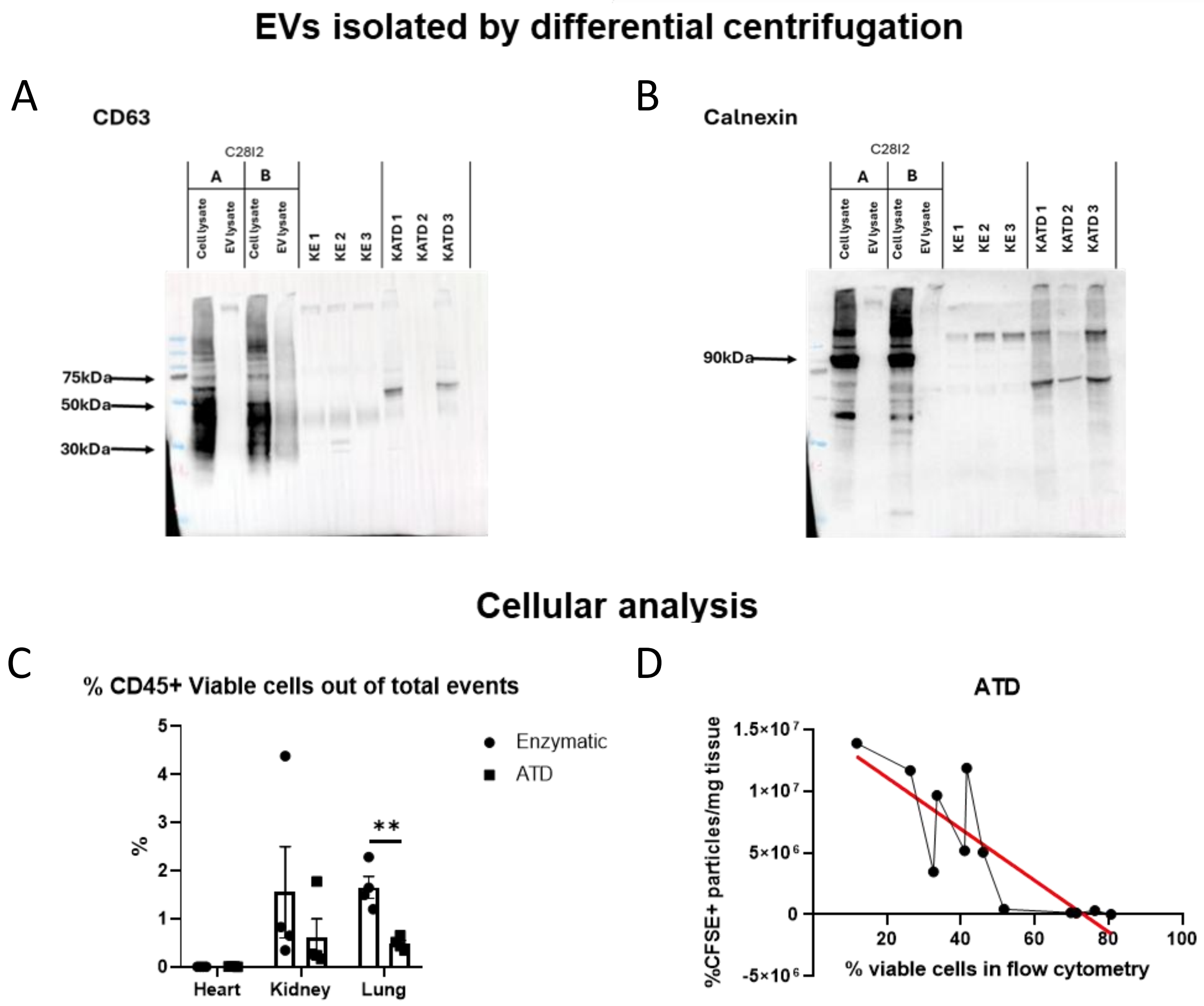

Supplemental Table 1

Date	Animal #	Weight lung total	Weight heart total	Weight kidney total	Weight lung enzymatic	Weight lung tissue grinder	Weight heart enzymatic	Weight heart tissue grinder	Weight kidney enzymatic	Weight kidney tissue grinder
29_8_24	1	330.1	163.6	193.1	132.4	193.8	77.6	85.3	98.5	83
29_8_24	2	322.3	136.6	213.5	132.8	140.5	69.3	67.5	97	104.1
29_8_24	3	255.4	149.5	190.8	128.7	105.8	70.15	70.6	84.5	93.1
29_8_24	4	248.3	144.5	176.2	102.5	120	69.7	64.4	76.6	87.2

Date	Animal #	Weight lung total	Weight heart total	Weight kidney total	Weight lung enzymatic	Weight lung tissue grinder	Weight heart enzymatic	Weight heart tissue grinder	Weight kidney enzymatic	Weight kidney tissue grinder
2022	1	213,4	132	380,6	107,6	105,8	69,6	62,4	191,8	188,8
2022	2	169,4	147,3	385,2	87,8	81,6	69,2	78,1	194	191,2
2022	3	219,7	169,3	437,3	107,9	111,8	78,2	91,1	224,7	212,6
2022	4	172,7	145,4	405,3	86,3	86,4	69,1	76,3	202	203,3
